# Closing the Critical Period Is Required for the Maturation of Binocular Integration in Mouse Primary Visual Cortex

**DOI:** 10.3389/fncel.2021.749265

**Published:** 2021-11-26

**Authors:** Jiangping Chan, Xiangwen Hao, Qiong Liu, Jianhua Cang, Yu Gu

**Affiliations:** ^1^State Key Laboratory of Medical Neurobiology, MOE Frontiers Center for Brain Science, Institutes of Brain Science, Fudan University, Shanghai, China; ^2^School of Life Sciences, Westlake University, Hangzhou, China; ^3^Department of Biology, University of Virginia, Charlottesville, VA, United States; ^4^Department of Psychology, University of Virginia, Charlottesville, VA, United States

**Keywords:** critical period, binocular matching, E/I balance, ocular dominance plasticity, orientation selectivity

## Abstract

Binocular matching of orientation preference between the two eyes is a common form of binocular integration that is regarded as the basis for stereopsis. How critical period plasticity enables binocular matching under the guidance of normal visual experience has not been fully demonstrated. To investigate how critical period closure affects the binocular matching, a critical period prolonged mouse model was constructed through the administration of bumetanide, an NKCC1 transporter antagonist. Using acute *in vivo* extracellular recording and molecular assay, we revealed that binocular matching was transiently disrupted due to heightened plasticity after the normal critical period, together with an increase in the density of spines and synapses, and the upregulation of GluA1 expression. Diazepam (DZ)/[(R, S)-3-(2-carboxypiperazin-4-yl) propyl-1-phosphonic acid (CPP)] could reclose the extended critical period, and rescue the deficits in binocular matching. Furthermore, the extended critical period, alone, with normal visual experience is sufficient for the completion of binocular matching in amblyopic mice. Similarly, prolonging the critical period into adulthood by knocking out Nogo-66 receptor can prevent the normal maturation of binocular matching and depth perception. These results suggest that maintaining an optimal plasticity level during adolescence is most beneficial for the systemic maturation. Extending the critical period provides new clues for the maturation of binocular vision and may have critical implications for the treatment of amblyopia.

## Introduction

In the visual system, an individual binocular neuron in the primary visual cortex (V1) simultaneously receives and integrates visual inputs from the two eyes. Most of these V1 neurons are also selective for specific stimulus orientation through each eye, and their orientation preferences are binocularly integrated in adults (Hubel and Wiesel, 1962). It is well appreciated that during the critical period of early visual development, binocular neurons in V1 undergo tremendous circuit rewiring and rearrangement to form stable and reliable visual responses, under the guidance of balanced binocular visual experience (Wang et al., 2010; Gu and Cang, 2016). The asymmetry of the visual inputs across the two eyes during the critical period leads to reduced visual acuity and impaired stereopsis, which are the basic visual defects for amblyopia (Maconachie and Gottlob, 2015). Amblyopia can also cause many other visual impairments, such as the binocular mismatching of the orientation preference from the two eyes. There are accumulating pieces of evidence revealing that critical period plasticity is a key driver of the binocular matching process in visual cortical neurons under normal development (Wang et al., 2013; Levine et al., 2017; Mazziotti et al., 2017).

Binocular matching of orientation preference matures during the critical period for ocular dominance (OD) plasticity and is strongly dependent on visual experience (Wang et al., 2010; Gu and Cang, 2016; Levine et al., 2017; Hao and Gu, 2020; Li and Gu, 2020). Indeed, before critical period onset, individual neuron shows different preferred orientations between the two eyes. Orientation preferences for the two eyes gradually match during the critical period, and eventually show a similar preference in adults (Mazziotti et al., 2017). Monocular deprivation (MD) and dark exposure (DE) during the critical period can severely disrupt the binocular matching, while reactivating V1 plasticity after MD by environmental enrichment (EE) in adulthood can fully rescue the disrupted binocular matching (Levine et al., 2017). The disruption of binocular matching process by MD cannot naturally recover after the critical period, indicating that it requires the normal visual experience in the critical period. Indeed, this phenomenon is restricted to juveniles, neither MD nor DE in adult mice can affect binocular matching (Wang et al., 2013; Levine et al., 2017).

Interestingly, if the initiation of the critical period and binocular matching are intentionally uncoupled, the binocular matching process would be suppressed. For example, either overexpressing brain-derived neurotrophic factor (BDNF) or knocking out Mecp2 in mouse V1, which can induce a precocious opening and closure of the critical period, the binocular matching cannot be completed (Wang et al., 2013; Krishnan et al., 2015). Furthermore, if the mice with BDNF overexpression (BDNF-OE) are reared in an enriched environment to also advance the onset of the binocular matching process and match the precocious critical period onset, the binocular matching process can be rescued. Early EE or administration of Insulin-like Growth Factor 1 (IGF-1) consequently advances the matching process to coincide with the precocious plasticity and completely rescues the deficit (Ciucci et al., 2007; Wang et al., 2013). This suggests that the completion of binocular matching requires plasticity and circuitry rewiring during the whole critical period. However, it is not known whether the closure of the critical period plays an important role in the binocular matching process. If the critical period is not closed at the proper time point, three scenarios can happen: (1) the binocular matching can normally complete since it is already properly initiated and developed through the whole critical period; (2) the binocular matching cannot complete since it requires an active signal to drive its completion at the end of the critical period; (3) the binocular matching would remain mismatched until the closure of the extended critical period.

γ-gamma-aminobutyric acid plays a depolarizing role in the early developmental process (until the first/second postnatal week in rodents), which is the basis for the early physiological maturation of various brain areas. A critical period prolonged rat model revealed that interference with depolarizing γ-gamma-aminobutyric acid (GABA)ergic neurons during early development, induced by administrating bumetanide, an NKCC1 cotransporter inhibitor (Wang and Kriegstein, 2008; Liu et al., 2019), could protract the critical period for OD plasticity for about 10 days (Deidda et al., 2015). In this study, we established a critical period prolonged mouse model with bumetanide, and examined the progress of difference in orientation preference of two eyes at different stages of development, and the impact on visual function and morphological development of cortical neurons, through *in vivo* single-unit electrophysiological recording and molecular and cellular assay. Our results showed that binocular matching was transiently postponed in bumetanide treated (Bum) mice at P35 due to heightened plasticity in a specific time window after the normal critical period, underlying that the maturation of binocular matching is dependent on the proper closure of critical period. This result was accompanied by an increase in the density of dendritic spines and synaptic markers, upregulation of GluA1 expression, and a reduction of extracellular matrix perineuronal nets (PNNs). Pharmacological administration of diazepam (DZ)/[(R, S)-3-(2-carboxypiperazin-4-yl) propyl-1-phosphonic acid (CPP)] could reclose the extended critical period and rescue the deficits in binocular matching. In addition, the 10-day extended critical period alone with normal binocular visual experience is sufficient to rematch the completely mismatched orientation preference in the mice with chronic MD (amblyopia). Furthermore, knocking out nogo-66 receptor, which prolonged the critical period into adulthood (McGee et al., 2005), could suppress the normal completion of binocular matching and the maturation of depth perception in the NgR1^–/–^ mice. These data indicate that maintaining an optimal plasticity level during adolescence is most beneficial for the systemic maturation. Either too low or too high of the plasticity at different time points may cause deficits in the development of binocular integration.

## Materials and Methods

### Animals

The constitutive NgR1 mutant mice (NgR1^–/–^) (Stephany et al., 2016, 2018) were generous gifts from Dr. Aaron W. McGee (University of Louisville). The NgR1^–/–^ and wild-type C57BL/six mice were housed at 24°C, in a 12 h light, 12 h dark cycle, and given food and water available *ad libitum*. Mice were housed with four or five littermates. Experiments were performed on both male and female mice. All efforts were made to minimize animal discomfort. All protocols and procedures followed the guidelines of the Animal Care and Use Committee of Fudan University.

### Drug Administration

The mice were injected with bumetanide (0.4 mg/kg; Abcam), 7,8-dihydroxyflavone (DHF, 10 mg/kg; Abcam), or dimethyl sulfoxide (DMSO, 0.04% in saline) intraperitoneally (*i.p.*) twice a day from P3 to P8, as described previously with some modification (Deidda et al., 2015). Treated mice at different ages (P21, P28, P35, and P45) were used in the experiments. The competitive N-methyl-d-aspartate (NMDA) receptor antagonist [(R, S)-3-(2-carboxypiperazin-4-yl) propyl-1-phosphonic acid (CPP, Tocris Bioscience)] was dissolved in saline at a concentration of 1.5 mg/ml, and the solution or the same volume of saline was injected (*i.p*.) at a dose of 15 mg/kg every ∼4 h starting from P31. CPP was administered from P31 for 4 consecutive days before 4d MD. The CPP data were compared to Bum or Veh, without further control experiment.

### Cannula Fixation and Diazepam Injection

Mice were placed in a plastic chamber, anesthetized with isofluorane in air (5% for induction, 1–2% for maintenance), and then placed in a stereotaxic apparatus (RWD Life Science) and their scalp were shaved and disinfected with 75% ethanol. Ocular lubricant was used to protect the eyes of the animals from drying during surgery. A skull drill was used to perform a small craniotomy above the right lateral ventricles (AP: –0.6 to –0.7 mm; LM: –1.45 to –1.5 mm; DV: –1.7 to –1.8 mm), and a 27G stainless-steel cannula with a plastic cup (RWD Life Science) was affixed to the bone using dental cement. A matched obturator cap was used to seal the cannula. The animals were then maintained at approximately 37°C on an electric heating blanket and housed separately until complete recovery from anesthesia. Then, animals were allowed to recover for at least 3 days prior to infusions. In addition, an osmotic minipump (Longer Pump, 750 nl/min) containing diazepam (1.5 μl/side, 2 mg/ml in saline solution) through a syringe (Chen et al., 2014) was connected to cannula into the right lateral ventricles. After the injection, the syringe remained in the brain for 10 min to allow for diffusion of the drug. DZ was administered from P31 for 4 consecutive days before 4d MD. The DZ data were compared to Bum or Veh without any further control experiment.

### Monocular Deprivation

Animals were anesthetized with isofluorane in air (5% for induction, 1–2% for maintenance). The margins of the upper and lower lids of one eye were trimmed and sutured together with three mattress sutures (6-0 silk, Ethicon) (Cang et al., 2005; Baroncelli et al., 2013). The animals were returned to their home cages until they were alert and mobile. Erythromycin ointment was applied after surgery to prevent inflammation of the wound. The duration of eye closure was 4 days or long-term monocular deprivation (LTMD, ∼15 days). A subset of mice treated postnatally with bumetanide (Bum) and vehicle (Veh) were MD at three different ages: deprivation was initiated P25, P35, and P45, respectively. For rescue experiment, Bum and Veh mice were MD from P35 for 4 days. For the LTMD experiment, mice were MD during the critical period for OD plasticity (P19-P35) by eyelid closure. Eyes were checked every 2–3 days for proper closure. Eye health was further monitored for ∼10 days (until P45) following eye reopening. The suture was removed with fine scissors and examined under a stereomicroscope before electrophysiological recordings. Mice with scarring of the cornea and signs of infection were eliminated from the study.

### *In vivo* Electrophysiology

Mice were anesthetized with urethane (20 mg/kg, *i.p.*, Sigma-Aldrich) and then sedated with chlorprothixene (5 mg/kg, intramuscular [*i.m.*], Sigma-Aldrich) (Wang et al., 2010). The animal was placed on a stereotaxic apparatus and maintained at 37°C by a feedback-controlled heating pad (Harvard Apparatus). Silicon oil was applied to both eyes to prevent them from drying. A small craniotomy (∼1.5 mm^2^) was made on the left hemisphere to expose binocular zone of visual cortex (V1b, 3.0–3.3 mm lateral from the midline and 0.5–0.8 mm anterior from the lambda suture). A linear silicon electrode (16 channels, ASSY-1-16-1, Lotus Biochips, United States) was penetrated perpendicular to the pial surface in the binocular zone. For each animal, two to five penetrations were made with minimum spacing of 80μm and cells recorded across all layers (from the surface to a depth of 700 μm) were included in our recordings (Levine et al., 2017). Signals were acquired using OmniPlex Neural Recording Data Acquisition System (Plexon Inc., Dallas, United States). Electrical signals were filtered from 0.3 to 6 kHz for spike, and 0.5–300 Hz for visual evoked potential (VEP). Sampling rate was 40 kHz. Throughout recordings, toe-pinch reflex was monitored and additional urethane was supplemented as needed. Two homemade tapes were put close to individual eyes and alternately open to achieve monocular visual stimulation during recordings. At the end of each recording, the animal was sacrificed for molecular and morphological experiments.

### Visual Stimuli

Visual stimuli were generated with Psychopy v3.0 and presented on a monitor with gamma correction (Dell, 57 × 34 cm, 60 Hz refresh rate, ∼100 cd/m^2^ luminance) placed 20 cm directly in front of the animal. All measurements were performed in the primary binocular visual cortex. To measure orientation selectivity, the direction of the drifting sinusoidal gratings varied between 0 and 330° (12 steps at 30° spacing) in a randomized sequence. The spatial frequency of the stimuli was 0.04 c/deg and the temporal frequency was 2 Hz. Meanwhile, the stimulus was presented with a nominal 100% contrast. Each stimulus was presented for 1.5 s, with 1.5 s inter-stimulus gray screen interval. A gray blank condition (mean luminance) was included to estimate the spontaneous firing rate. For each recording session, the sequence was repeated for 5 trials. For VEP recording, stimuli were horizontal square gratings of different spatial frequency (0.04, 0.08, 0.16, 0.32, 0.64 c/deg) in pseudorandom order and 100% contrast while temporal frequency was also 2 Hz. The blank screen stimulation is used as control.

### Visual Cliff Test

For NgR1^–/–^ and wild-type (WT) mice, the visual cliff behavior was examined in an open-top acrylic box measuring 60 × 45 × 60 cm, which was placed on the edge of a laboratory bench so that half of the box was on the bench (safe side) and the other half was 85 cm above the floor (cliff side). A black-and-white-striped pattern was displayed on the countertop and the floor below. A circular plastic platform (9.6 cm diameter × 2 cm) was stationed in the middle of the box equally intersecting both the safe side and the cliff side (Han et al., 2017). For Bum and Veh mice, the visual cliff apparatus consisted of a rectangular (54 × 36 × 36 cm) acrylic board with white paper walls. The area was divided into two zooms of equal size with a long black bar. A patterned floor consisting of 3 × 3 cm red and white checked photographic paper covered the surface of the platform. On the shallow side, the patterned floor is placed directly under the glass plate, while the distance on the deep side is 60 cm. Incandescent lamps placed below the patterned floors illuminate both surfaces to equate the brightness of the two sides (Mazziotti et al., 2017). The test was conducted in a quiet room. A tele-camera was hanging on the apparatus and was connected to a computer by which the experimenter could observe and record the behavior of the animal. We trimmed the whiskers of the animals 0.5 h before each trial to ensure the engagement of the visual sensory system. Each mouse was placed on the platform and observed for 5 min beginning from its placement on the platform. Among animals, the shallow and deep positions of the arena are random. Each mouse received a single trial. The box and platform were cleaned with 75% alcohol after each trial. We use ToxTrac software^1^ for analysis. The arena was cleaned between trials with a 75% alcohol solution. In experiments, adult wild-type mice and MD mice were used to successfully verify the effectiveness of these two experimental devices (data not shown).

### Fluorescent Immunostaining and Analysis

Mice were anesthetized by injecting with an overdose of urethane (20 mg/kg, *i.p.*) and transcardially perfused with ice-cold saline, followed by 4% paraformaldehyde (PFA) in PBS. Brains were extracted and post-fixed in 4% PFA overnight at 4°C, and then cryprotected with 30% sucrose in 0.1M PBS for 2 days. 20-μm-thick coronal sections were cut with a cryostat microtome (Leica CM 1956, Germany) and stored in antifreeze protection fluid at –20°C.

### Synaptophysin and PSD-95 Puncta Staining

Brain sections were washed three times for 10 min each in PBS, then blocked for 2 h in 5% goat serum (Gibco, 2079017) and 0.2% Triton X-100 (Solarbio, T8200) at room temperature (RT). Then the sections were incubated with the primary antibodies (synaptophysin 1:500, Synaptic systems; PSD-95 1:800, Abcam) overnight at 4°C. On the second day, the sections were rinsed with PBS and incubated with the secondary antibody (donkey anti-mouse Alexa Fluor 674; donkey anti-rabbit Alexa Fluor 488; Jackson) for 2 h at RT. Images of the brain sections containing layer IV/V of V1b were acquired using a 60 × NA1.4 immersion objective lenses on confocal microscopy systems (Olympus, FV1000). Images were taken at a resolution of 1,024 × 1,024 and at zoom 3 to quantify the density of synaptophysin and PSD-95 puncta. Z stacks were acquired with a 1μm step and compressed the strongest middle three or four z-sections to generate the final image. Stacks were exported as TIFF files and analyzed using Image-Pro Plus (Media Cybernetics). HiGauss filter was applied to each image to enhance the fine details and maximize the total number of puncta obtained from each image. The mean puncta number per 1,000 μm^2^ was used as an estimate of the density of synaptic puncta (Abumaria et al., 2011; Liu et al., 2018).

### Perineuronal Net Staining

For PNN labeling, free floating sections were washed in PBS and incubated for 2 h in a blocking solution (3% BSA in PBS for WFA staining) at RT followed by incubation with the appropriate antibodies. The sections were then incubated overnight at 4°C with Lectin from Wisteria floribunda (WFA 1:200, Sigma). After primary antibody incubation, the sections were washed in PBS followed by secondary antibody incubation with Cy3 labeled streptavidin (Sangon Biotech). Each IHC staining round was accompanied by nuclear cell counterstaining with 4′,6-diamidino-2-phenylindole (DAPI 1:1,000, Solarbio) in PBS. All slides were mounted with anti-fading Fluoromount™ (Sigma, F4680) and left at 4°C in a dark chamber until imaging. The brain sections were acquired using identical parameters with fluorescent microscopy (Nikon Eclipse Ni; Olympus VS120) and V1b were identified according to Mouse Brain Atlas. Five z sections were stacked for each image, exported as TIFF files. The number of WFA-positive neurons in V1 binocular zone was counted by Image J in an area of 500 × 550 μm. The averaged cell density of each mouse was compared between the experimental groups.

### DiI Labeling

Mice were anesthetized by injecting with an overdose of urethane and transcardially perfused with ice-cold saline, followed by 1.5% PFA in PBS. Brains were extracted and post-fixed in 1.5% PFA for 1 h and transferred to 0.1M PBS (PH7.4). Then, coronal sections (100 μm) were cut using Leica vibratome (Leica VT 1200s, Germany). With the aid of a thin histological needle, the sonicated fine powdered lipophilic dye dialkylcarbocyanine (DiI, thermo, D282) was gently placed over the tissue. The exposed tissue sections were kept on glass slides covered with PBS at room temperature for approximately 12 h in the dark. After that, the 0.1M PBS was removed and the tissue was finally fixed with 4% PFA in PBS for 30 min. The fixation solution was rinsed with 0.1M PBS and coverslipped with Fluoromount™, avoiding air bubbles. To prevent the mounting media to dry, the borders of the coverslip were sealed with a fast-drying nail polish. Confocal imaging was done immediately after staining and the slides were refrigerated at –20°, preferably in no more than 2 days (Cheng et al., 2014). Apical and basal dendritic of V1b were randomly selected for imaging DiI fluorescence. Dendrites were randomly imaged using 60 × NA1.4 water immersion objective lenses on confocal microscopy systems (Nikon) at 3 × zoom and 1,024 × 1,024-pixel resolution. The spectral detectors were adjusted to capture emission from a helium/neon laser at wavelengths of 594 nm for DiI staining. The Z stack acquisitions were performed with fluorescence z-stacks of 9–10 μm thickness consisting of sections at 0.5 μm increments and rapidly scanned within an imaging area. The image acquisition was set at a range of 8 bits. Spine densities were calculated by quantifying the number of spines per 10 μm of dendritic length. For each group, apical and basal dendrites, mainly from layer IV/V of V1b, were quantified separately. Dendritic length and number were measured using Image J software.

### Golgi Staining

Experimental animals were deeply anesthetized with urethane and the brains were retrieved from the skull as quickly as possible without damage. Golgi-Cox staining to obtain visual cortical spines was conducted with the FD Rapid GolgiStain Kit (FD Neuro Technologies, Inc., Columbia, MD, United States) according to the instructions of the manufacturer with some modifications (Das et al., 2013). Coronal tissue sections of 150 μm were cut with leica vibrotome (Leica VT 1200s, Germany) at RT. The sections were mounted on gelatin-coated microscope slides (FD, P0101) using Solution C with brush. After drying naturally, the slides were immersed with solution D/E for 10 min. The slides were dehydrated in sequential rinses of 50, 75, and 95% ethanol for 4 min per rinse, 100% ethanol four times for 4 min per rinse, and cleared in xylene three times for 4 min per rinse. The sections were slip-covered with mounting medium (Sinopharm, 10004160). Golgi stained and mounted brain sections were imaged using 60 × NA1.42 oil immersion objective lenses on confocal microscopy systems (Nikon). Images of layer IV/V of V1b with 60 × lenses were taken at zoom 3 to quantify spine density. Z-section stack of images was taken at steps of 0.5 μm to generate the final image. The images were analyzed by Image J software and the density was estimated per 10 μm dendritic length.

### Western Blot

Experimental animals in each group were deeply anesthetized with urethane (20 mg/kg, *i.p.*) and decapitated after the mice had lost their pain response. The bilateral visual cortices were dissected and mixed into one sample, immediately frozen, and then stored at –80°C until processing. Proteins were extracted with lysis buffer (RIPA lysate: PMSF = 100:1, Beyotime). Rude lysate by ultrasonic was then centrifuged at 13,000 rpm at 4°C for 10 min. The supernatant contained the total protein lysate, and protein concentrations were measured using the BCA assay kit (Thermo, 23225). Protein extracts (10–50 μg per lane, depending on the protein) were boiled for 10 min at 100°C and then loaded on homemade gels, separated using sodium dodecyl sulfate polyacrylamide gel electrophoresis (SDS-PAGE, 30 min at 80 V and 1 h at 120 V), and blotted on PVDF membrane (Millipore, IPVH00010, 0.45 μm). The membranes were blocked using a solution of 10% no-fat milk, dissolved in 0.2% Tween-20 in Tris-buffered solution (TBS) for 2 h at RT and then incubated overnight at 4°C in 5% milk dissolved TBST with the following primary antibodies: NKCC1 (mouse monoclonal, #T4, 1:500, DSHB), BDNF (rabbit polyclonal, 1:1,000, Protech), AMPA Receptor 1 (GluA1, rabbit monoclonal, 1:1,000, Cell Signaling Technology), GABA_A_ Receptor α1 (rabbit polyclonal, 1:500, Millipore), GRIN2A (rabbit polyclonal, 1:2,000, ABclonal), and GRIN2B (rabbit polyclonal, 1:2,000, ABclonal). The membranes were then incubated with 1:2,000 horseradish peroxidase (HRP)-conjugated secondary antibodies for 2 h at RT to reveal the signal in chemiluminescence agent (Beyotime) using the enhancer system (Bio-Rad ChemiDoc Touch Imaging System). As an internal standard, all blots were probed for GAPDH (mouse monoclonal, 1:2,000, Abcam). Western blot needed to be repeated at least three times, and the light density of each strip was measured by image J. All protein levels were normalized to GAPDH levels. Measurement and quantification were always performed blind for the experimental groups.

### Electrophysiological Data Analysis

Single units were identified using Offline Sorter (Plexon) by principal component analysis which was performed to score spikes with a high degree of similarity in a 3D feature space and Neuroexplorer (Plexon) for follow-up processing. Data analysis was further performed using a custom software written in python. For spikes data, average number of spikes during the 1.5 s stimulation were calculated as response magnitude (R) with the spontaneous rate subtracted to obtain the response to a particular stimulation condition across all trials. The responses at the direction of θ, R(θ), were used to calculate gOSI = ΣR(θ)e^2*i*θ^ /ΣR(θ) which is the normalized vector sum of the responses in the 180° orientation space. Its amplitude was used as a global orientation selective index (gOSI), which is the same as 1-circular variance. Half of its complex phase was calculated and then converted to the preferred orientation (pref_O) by subtracting 90° to confine pref_O between –90 and 90°. The difference in the preferred orientation between the two eyes was calculated by subtracting ipsilateral pref_O from contralateral pref_O along the 180°cycle, and differences > 90° would be subtracted from 180° to convert them to values between 0 and 90°. The absolute value of two eyes difference was used in all quantifications (Wang et al., 2010; Levine et al., 2017; Mazziotti et al., 2017). All data are tested for normality before analysis, and the cells were divided into cells with high orientation selectivity and cells with low orientation selectivity with a cut-off at 0.4. Units were then classified as narrow or broad spiking neurons on the basis of properties of their average waveforms by means of spike width-based (trough-to-peak time) (Niell and Stryker, 2008). Only binocular cells were included in our analysis, the degree of ocular dominance was quantified by an index, ODI = (C-I)/(C + I), where C and I represent the magnitude of the spikes caused by the visual stimulus presented to the contralateral or ipsilateral eye after the spontaneous response is subtracted. The ocular dominance index (ODI) ranges from + 1 to –1, where 1 indicates a contralateral bias, and –1 an ipsilateral bias (Sato and Stryker, 2008). Units were classified by classic seven-point scheme (Hubel and Wiesel, 1962; Gordon and Stryker, 1996). Correspondingly, the contralateral bias index (CBI) was then calculated according to the following formula: CBI = [(N1-N7) + (2/3) *(N2-N6) + (1/3) *(N3-N5) + N]/2N, where Nx was the numbers of cells with ocular dominance category equal to x and N was the total number of examined cells. All units were also scored for noise and response strengths on a 1 (weak noise/response firing) to 3 (strong noise/response firing) (Cai et al., 2017). On this basis, ∼30% of recorded units were discarded of the analysis. For local field potential, binocular VEPs were examined to measure visual acuity, which was assessed using different spatial frequency (0.04, 0.08, 0.16, 0.32, 0.64 c/deg) in pseudorandom order. VEPs in response to a blank stimulus were recorded to estimate the noise level. For each animal, VEP amplitude was plotted as a function of log spatial frequency and visual acuity was determined by linearly extrapolating VEP amplitude to 0 V (Beurdeley et al., 2012). For the interspike interval (ISI) analysis, we examined the distribution of the firing interval of all adjacent action potentials in a single neuronal firing sequence, and used the interval time as the X axis and frequency as the Y axis, expressed in the form of histograms. Since all the firing intervals of the neuronal firing sequence are greater than zero, the X-axis of the firing interval histogram has only positive values. Specifically, we first set a time window (bin), and marked the first time window as [a, a + bin], and the second time window as [a + bin, a + bin*2], and so on. The discharge interval greater than the left time scale of the time window was included in this time window, and the discharge interval greater than the right time scale was not included in this time window. Finally, for each time window, we calculated the total number of firings in the entire single neuronal firing sequence whose firing interval fell within the time window. The firing interval analysis can reflect the frequency and overall distribution of different firing intervals of the neuron (Delescluse and Pouzat, 2006; Chen and Nitz, 2011).

### Statistical Analysis

Statistical analyses were performed using GraphPad Prism software (version 8.0) and custom python scripts. All data were tested for normality before analysis (by Shapiro–Wilk test) and statistical testing was performed accordingly. Differences between two groups were assessed with the Student’s unpaired *t*-test for normally distributed data or with the Mann-Whitney rank-sum test for non-normally distributed data. Differences between multiple groups were assessed with one-way ANOVA or two-way ANOVA. *p*-values of < 0.05 were considered significant. Data were expressed as mean ± SEM except for box charts.

## Results

### Early Bumetanide Interference Prolongs Critical Period Plasticity in Mouse V1b

**FIGURE 1:**
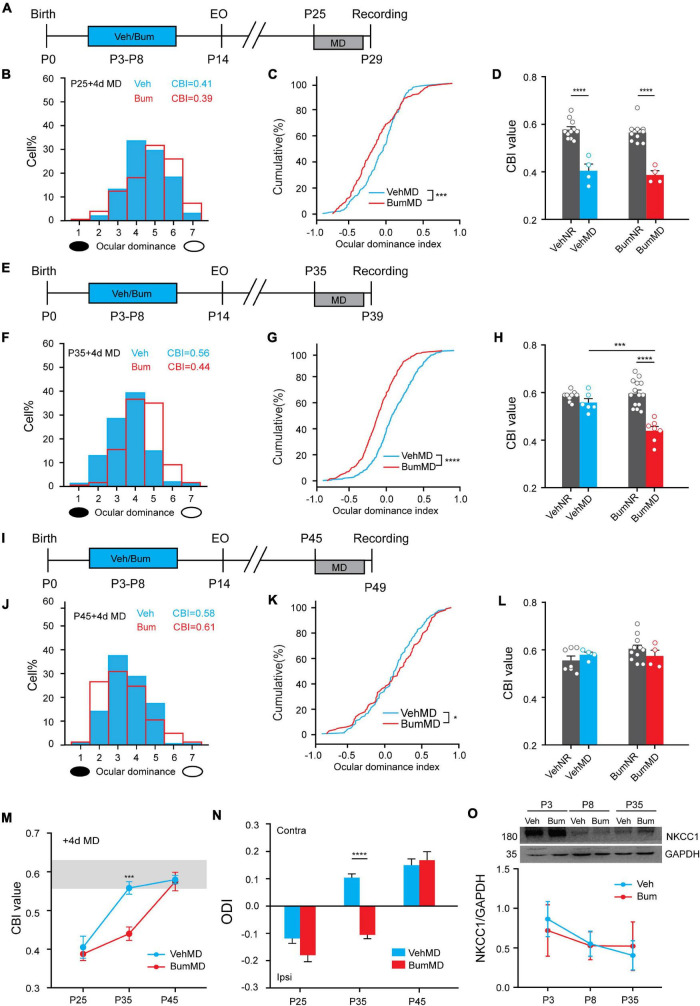
Early bumetanide interference prolongs critical period plasticity in mouse binocular zone of visual cortex (V1b). **(A)** Contralateral eye bias assessed by single-unit electrophysiology in mouse primary visual cortex after monocular deprivation for 4 days (4d MD) at P25. **(B)** Ocular dominance (OD) distribution for vehicle (Veh, blue) and bumetanide (Bum, red) were similar. The OD distribution is still significantly shifted toward the non-deprivation eye (open circle) for Veh and Bum mice. **(C)** OD cumulative distribution for Veh and Bum group. OD distribution for Bum group (177 cells, four mice) statistically different from Veh mice [295 cells, four mice, *p* < 0.0004, Kolmogorov-Smirnov (K-S) test]. **(D)** Contralateral bias index (CBI) value for VehMD, VehNR, BumMD, and BumNR animals. The CBI of VehNR mice was significantly different from that of VehMD animals (VehNR: 166 cells, 11 mice, *p* < 0.0001); The CBI of BumNR mice was significantly different from that of BumMD animals (BumNR: 159 cells, 10 mice, *p* < 0.0001, One-way ANOVA with Tukey’s *post hoc* test). **(E)** The same as **(A)** for P35. **(F)** The OD distribution is significantly shifted toward the non-deprived eye with respect to that in Veh animals. **(G)** OD cumulative distribution for Veh and Bum groups. OD distribution for Bum group(374 cells, seven mice)statistically different from Veh mice (463 cells, six mice, *p* < 0.0001). **(H)** The same as for **(D)**. The CBI of VehMD animals (VehMD: 463 cells, 6 mice) was not different from VehNR animals (VehNR: 102cells, 9 mice, *p* = 0.7021); The CBI of BumNR mice was significantly different from BumMD animals (BumMD: 374 cells, 7 mice; BumNR: 142 cells, 14 mice, *p* < 0.0001); The CBI of VehMD mice was significantly different from BumMD animals (*p* = 0.0002). **(I)** The same as for **(A,E)**. **(J)** The OD distribution is not different from the OD distribution observed in Veh animals without OD shift. **(K)** OD cumulative distribution was similar between two groups (BumMD: 164 cells, four mice; VehMD: 184 cells, four mice, *p* = 0.022). **(L)** The same as for **(D,H)**. The CBI of VehNR mice was not different from that of VehMD animals (VehNR: 96 cells, seven mice, *p* = 0.47); The CBI of BumNR mice was significantly different from that of BumMD animals (BumNR: 145 cells, 11 mice, *p* = 0.27). **(M)** Mean CBI value of Bum and Veh mice P25, P35, P45 with 4d MD, respectively (P35: Veh vs. Bum, *p* = 0.00012, Two-way ANOVA with Tukey’s *post hoc* test). The gray box indicates the typical range of CBI values for non-deprived mice. **(N)** OD index following brief 4d MD of the contralateral eye at P25\P35\P45 in Veh and Bum mice. MD induces a larger OD shift in Bum mice than in Veh littermates at P35 (*p* < 0.0001, One-way ANOVA with Tukey’s *post hoc* test). Y-axis shows OD index as described in Methods. **(O)** Cropped images and quantifications of immunoblotting for NKCC1 on protein extracts from P3\P8\P35 visual cortices of Veh and Bum. GAPDH was used as the internal standard (P3: *p* = 0.96; P8: *p* = 0.99; P35: *p* = 0.98, four mice per group; Two-way ANOVA with Tukey’s *post hoc* test). A black ellipse under the histogram indicates deprived eye. Open circles represent individual CBIs for each animal. CBI, contralateral bias index; ODI, ocular dominance index. A positive ocular dominance index (ODI) indicates contralateral (contra), a negative ODI ipsilateral (ipsi) dominance. Data were expressed as mean ± SEM. *p < 0.05, ***p < 0.001, ****p < 0.0001.

Previous studies have shown that brief Bum interference with depolarizing GABA during early development prolonged critical-period plasticity in visual cortical circuits without affecting the overall development of visual system in rats (Deidda et al., 2015). Here, we want to explore whether the same pharmacological manipulations on mice can also prolong the critical period. In order to answer this question, we used a classic paradigm of experience dependent plasticity—monocular deprivation (MD). We treated mice pups with the NKCC1 inhibitor bumetanide (i.e., 0.4 mg/kg body weight, twice a day) from P3 to P8 using protocol from Deidda et al. (2015) with some modification (Figure 1A). To define the time course of the critical period for OD plasticity, we performed 4d MD starting from P25 (peak of normal critical period), P35 (closure of normal critical period), P45 (presumably the end of the extended critical period), respectively. After 4d MD, we assessed OD distribution by single-unit recordings from the binocular field of the primary visual cortex contralateral to the deprived eye in Bum and Veh treated mice. OD was quantitatively attributed to each unit on the basis of the computer-acquired response to visual stimulation of either eye according to a modified Hubel and Wiesel classification (Hubel and Wiesel, 1970), and by assigning to each neuron, a normalized ocular dominance score. The OD distribution of both Veh and Bum animals after 4d MD significantly shifted toward non-deprived eye at P25 (Figure 1B). The changes of OD were confirmed by analyzing the cumulative distribution of the OD index (Figure 1C, K–S test, *p* < 0.001). Four-day MD was effective in shifting OD distribution in Bum mice at P35, but not in Veh mice (Figures 1E,F), and the distribution of Bum mice was statistically different from Veh animals (Figure 1G, K-S test, *p* < 0.0001). No OD shift in Bum or Veh mice were induced by 4d MD at P45 (Figures 1I,J), even though the distribution of Bum mice was slightly different from that of Veh animals (Figure 1K, K-S test, *p* < 0.05). Meanwhile, to strengthen the interpretation of the MD results, we also compared the OD plasticity to each age-matched group that were normally reared (NR) without MD. The OD distribution of the animals in each group was summarized with the contralateral bias index (CBI), in which 0 represents complete ipsilateral bias and 1 represents complete contralateral bias. Electrophysiological data indicated that in both Veh and Bum mice at P25, MD showed strong OD plasticity compared to NR (Figure 1D, VehMD: 0.41 ± 0.024; VehNR: 0.58 ± 0.01; BumMD: 0.39 ± 0.014; BumNR: 0.56 ± 0.01; VehMD vs. VehNR: *p* < 0.0001; BumMD vs. BumNR: *p* < 0.0001, one-way ANOVA with Tukey’s *post hoc* test). Conversely, consistent and significant OD plasticity was seen in Bum mice only at P35 with respect to aged-matched BumNR mice, while 4d MD in Veh group was insufficient to induce OD shift compared to age-matched VehNR mice (Figure 1H, VehMD: 0.56 ± 0.014; VehNR, 0.59 ± 0.014; BumMD: 0.44 ± 0.016; BumNR: 0.60 ± 0.014; VehMD vs. VehNR: *p* = 0.66; BumMD vs. BumNR: *p* < 0.0001). The CBI of Veh and Bum NR mice were indistinguishable from the values of two groups receiving MD at P45 (Figure 1L, VehMD: 0.58 ± 0.009; VehNR: 0.56 ± 0.017; BumMD: 0.61 ± 0.02; BumNR, 0.60 ± 0.015; VehMD vs. VehNR: *p* = 0.85; BumMD vs. BumNR: *p* = 0.72). Our CBI value in control and MD animals were consistent with previous literature (Pizzorusso et al., 2002; Harauzov et al., 2010). We also directly compared mean OD index after 4d MD between Bum and Veh mice at different ages (Figure 1N). At P25, Veh and Bum mice subjected to 4d MD both showed great plasticity. There is no difference in OD index between the two groups (VehMD: –0.12 ± 0.018; BumMD: –0.17 ± 0.024, *p* = 0.28, one-way ANOVA with Tukey’s *post hoc* test). At P35, Bum mice showed great plasticity after brief MD, with a significant lower OD index than age-matched Veh group (VehMD: 0.10 ± 0.014; BumMD: –0.11 ± 0.014, *p* < 0.0001). In both Veh (0.15 ± 0.023) and Bum (0.17 ± 0.031) mice at P45, OD remained biased toward the contralateral eye after 4d MD and was not significantly different between the two groups. These results showed that the critical period for OD plasticity was indeed extended for about 10 days (from P35 to P45) in Bum mice (Figure 1M, two-way ANOVA and Tukey’s *post hoc* test, *p* < 0.001), similar to the rat model (Deidda et al., 2015). We further explored the effect of early bumetanide treatment on the expression of NKCC1 protein. We found that the expression of NKCC1 was declined during the development, but the Bum group at different ages were similar to age-matched Veh group (Figure 1O), consistent with previous report (Deidda et al., 2015).

### Binocular Matching of Orientation Preference Was Disrupted in Mice With Extending Cortical Plasticity at P35

**FIGURE 2:**
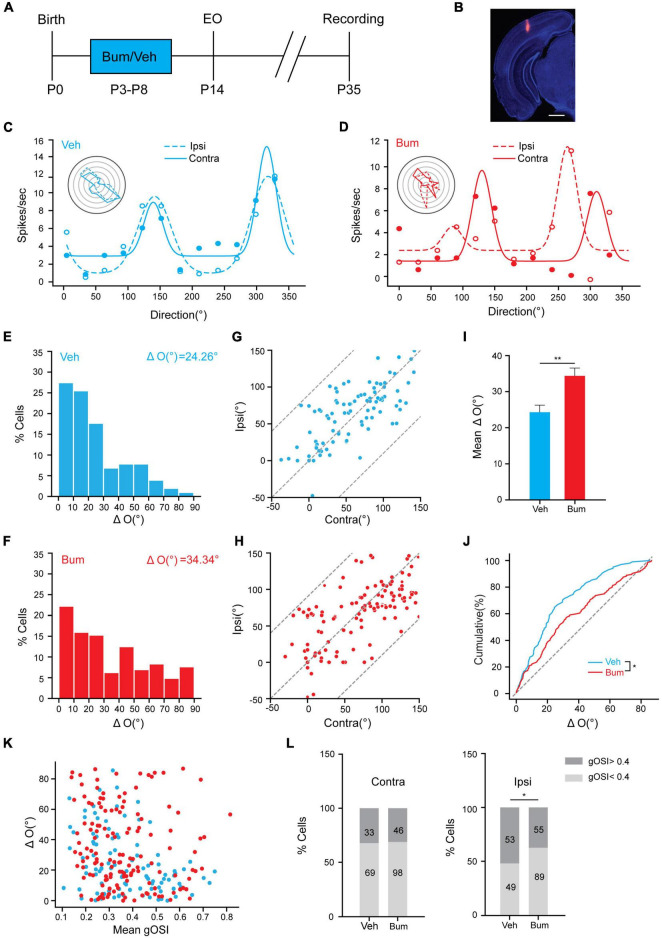
Binocular matching of orientation preference is impaired in Bum mice at P35.**(A)** Experimental paradigm. **(B)** Image showing traces of electrode in V1b. Red indicates electrode traces. Scale bar, 500 μm. **(C,D)** Orientation tuning curves of a visually responsive unit for **(C)** Veh and **(D)** Bum mice. Representative polar plots of orientation-tuned responses from contralateral (solid line) and ipsilateral (dashed line) eye are depicted. **(E,F)** Distribution of difference in preferred orientations through the two eyes in **(E)** Veh (ΔO = 24.26 ± 1.54°) and **(F)** Bum (ΔO = 34.34 ± 2.15°) mice. The data were binned at 10° from 0 to 90°. **(G,H)** Correlation of orientation preference between the two eyes. **(G)** High correlation binocularly (*r* = 0.69, *p* < 0.0001, 102 cells, 10 mice) in Veh mice at P35, with most points cluster close to the unity line. The dotted lines bound the region in which the data points can lie. **(H)** Correlation (*r* = 0.51, *p* < 0.0001, 142 cells, 14 mice) is low in Bum mice with respect to Veh group. **(I)** Mean ΔO is significantly higher in Bum than Veh animals (*p* = 0.0035, Mann-Whitney rank-sum test) at P35. **(J)** Cumulative distribution of ΔO for Bum and Veh mice at P35 (*p* = 0.014, K-S test). The ΔO distribution of random matching (dotted gray line) is shown for comparison. **(K)** Scatter plots of ΔO as function of orientation selectivity for individual cells (mean gOSI of the monocular tuning curves) for Veh (blue, *r* = 0.69, *p* = 1.06 × 10^–15^) and Bum mice (red, *r* = 0.51, *p* = 5.05 × 10^–11^). **(L)** The proportion of cells with high orientation selectivity and low orientation selectivity in the ipsilateral (right) (χ^2^-test, *p* < 0.05) or contralateral (left) eye, respectively, in Veh and Bum animals (dark gray: high selectivity gOSI > 0.4; light gray: low selectivity gOSI < 0.4). Data were expressed as mean ± SEM. *p < 0.05, **p < 0.01.

Precocious cortical plasticity induced by increasing inhibition is detrimental to the binocular matching of orientation preference (Wang et al., 2013). Thus, what impact does extending the critical period have on the binocular matching process? In order to answer this issue, we made single-unit recordings in the binocular zone of V1 in P35 Veh and Bum mice (Figures 2A,B) and determined monocular orientation tuning properties of individual neurons in response to drifting sinusoidal gratings of 12 orientations, respectively, for each eye. The monocularly preferred orientations were then compared between the two eyes, and their difference (“ΔO”) was used to quantify the degree of binocular matching. We found that the neurons were tuned to nearly identical orientations through the two eyes in Veh mice, but not in Bum mice (Figures 2C,D). In Bum mice, the difference of preferred orientation between two eyes (Figure 2F, ΔO = 34.34 ± 2.15°, 142 cells, 14 mice) was significantly higher than Veh controls (Figure 2E, ΔO = 24.26 ± 1.54°, 102 cells, nine mice, *p* = 0.0035). Such similarity in the preferred orientations between the two eyes was observed across the population in Veh mice (Figure 2G, correlation coefficient *r* = 0.69), which is higher than Bum mice (Figure 2H, *r* = 0.51). These results indicated that early bumetanide treatment specifically disrupted the matching of the two streams of eye-specific inputs in the cortex at a specific time while keeping monocular tuning properties intact (Figures 2I,J). It was previously shown that the development of binocular matching of orientation preference could be due to an increase in orientation selectivity of individual neurons (Wang et al., 2010). To better understand the relationship between the observed ΔO and orientation selectivity, we compared the level of matching in individual cells with the orientation selectivity of each cell (as quantified by global orientation selective index gOSI; see section “Materials and Methods” for details of calculation) in Veh and Bum mice. In Veh mice, cells with low gOSI showed ΔO values that spanned the entire 0–90° range, while cells with high gOSI had smaller ΔO. In Bum mice, on the contrary, the ΔO of cells with low and high gOSI both spanned the entire 0–90° (Figure 2K). Next, we asked if bumetanide treatment directly affected the gOSI for each eye. Intriguingly, the cells with higher orientation selectivity (gOSI > 0.4) in the ipsilateral eye were significantly decreased, while the cells with lower orientation selectivity (gOSI < 0.4) increased in Bum animals (Figure 2L, χ^2^-test, *p* < 0.05). This is consistent with a recent finding that the binocular matching is mainly driven by the sharpening of tuning from the ipsilateral input (Tan et al., 2020). These results suggested that bumetanide treatment may not only suppress the development of orientation selectivity in the ipsilateral eye, but also directly impaired the binocular matching in highly selective neurons.

### The Effect of Bumetanide Treatment on Binocular Matching Was Limited to P35

**FIGURE 3:**
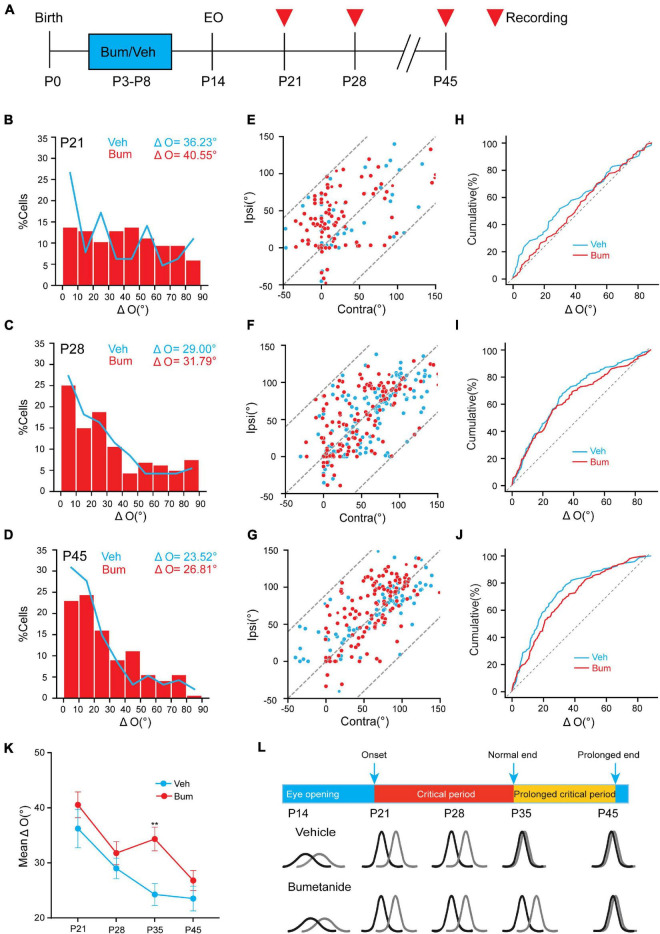
Bumetanide treatment has no effect on binocular matching at other ages except for P35. **(A)** Schematic of three age groups (P21, P28, P45) recorded in Veh and Bum animals. Red triangle indicates *in vivo* electrophysiological recordings. **(B–D)** Distribution of difference in orientation preference in three different ages in Veh and Bum groups: **(B)** P21 in Veh (blue, ΔO = 36.23 ± 3.46°, 65 cells,16 mice) and Bum groups (red, ΔO = 40.54 ± 2.33°, 116 cells, 18 mice); **(C)** P28: in Veh (ΔO = 29.00 ± 1.86°,165 cells, 11 mice) and Bum groups (ΔO = 31.79 ± 2.08°, 159 cells, 11 mice); **(D)** P45: in Veh (ΔO = 23.52 ± 2.24°, 94 cells, seven mice) and Bum groups (ΔO = 26.81 ± 1.80°, 143 cells, 11 mice); The data were binned at 5 ms from 0 to 1s. The same color pattern is followed in all figures. (**E–G)** The correlation of preferred orientation of two eyes. **(E)** P21: orientation preferences are highly uncorrelated binocularly in the two groups (Veh: *r* = 0.48, *p* < 0.0001; Bum: *r* = 0.55, *p* < 0.0001, K-S test), and most points are discretely distributed. The dotted line defines the area where the data points are located. **(F)** P28: the correlation was better than P21 (Veh: *r* = 0.69, *p* < 0.0001; Bum: *r* = 0.64, *p* < 0.0001, K-S test). **(G)** P45: orientation preferences are highly correlated in the two groups (Veh: *r* = 0.79, *p* < 0.0001; Bum: *r* = 0.69, *p* < 0.0001, K-S test), and most points are close to the unity line. **(H–J)** Cumulative distribution of ΔO between the two eyes in two groups **(H)** at P21, **(I)** P28, and **(J)** P45. **(K)** Mean ΔO of visual cortical neurons in Veh and Bum mice at different ages. Mean ΔO of bumetanide treatment is higher than Veh animals only at P35 (*p* = 0.0053, two-way ANOVA with Tukey’s *post hoc* test). **(L)** Binocular matching of orientation preference is established during the prolonged critical period under bumetanide treatment. The colored curve represents the orientation tuning of a single neuron. The orientation tuning properties are both immature at the onset of critical period in Veh and Bum mice. The orientation tuning curves of individual neuron of the two eyes are highly consistent in Veh group at the end of normal critical period. Bumetanide treatment postpone binocular matching until the end of the extended critical period. Black and gray solid lines represent contralateral and ipsilateral eye, respectively. Data were expressed as mean ± SEM. **p < 0.01.

It was previously reported that a precocious critical period induced by overexpressing BDNF could result in binocular mismatching (Wang et al., 2013), and a mismatching induced by juvenile MD can be maintained into adulthood if the critical period plasticity is not reactivated (Levine et al., 2017). To ask if bumetanide could also affect the initiation of the critical period, and whether the effect of bumetanide is transient or long-term, we investigated whether bumetanide treatment was able to influence binocular matching at other ages. We examined binocular matching of orientation preference in Veh and Bum mice at P21, P28, and P45, respectively (Figure 3A). Single-unit data revealed no significant difference in the preferred orientations between the two eyes at P21 in Veh (ΔO = 36.23 ± 3.46°, 65 cells, 16 mice) and Bum (ΔO = 40.55 ± 2.32°, 116 cells, 18 mice, Figure 3B). There was no strong correlation between two eyes in bum and Veh mice (Veh: *r* = 0.48; Bum: *r* = 0.55), and distribution of ΔO of Bum was similar to age matched Veh (Figures 3E,H). As the development processed, the degree of mismatching compared to the beginning of the critical period were reduced at P28 (Figure 3C, Veh: ΔO = 29.00 ± 2.04°, 165 cells, 11 mice; Bum: ΔO = 31.79 ± 1.86°, 159 cells, 11 mice), and were still equivalent between the two groups at the peak of critical period. The degree of mismatch between the two eyes remained high in two groups (Figures 3F,I, Veh: *r* = 0.69; Bum: *r* = 0.64). After the closure of the extended critical period (P45), the orientation preference of visual cortical neurons in both groups were closely matched between the two eyes which reached adult level (Figures 3D,J, Veh: ΔO = 23.52 ± 2.25°, 94 cells, seven mice; Bum: ΔO = 26.81 ± 1.8°, 143 cells, 11 mice). There was a strong correlation between two eyes in Bum and Veh mice at P45 (Figure 3G, Veh: *r* = 0.79, Bum: *r* = 0.69). Remarkably, the degree of binocular mismatching between the two eyes were significantly high at P35 in Bum mice compared to age matched Veh groups (Figures 3K,L, two-way ANOVA and Tukey’s *post hoc* test, *p* = 0.0053). In other words, the binocular matching between the two groups were similar throughout development, with the only exception at P35. Together, binocular matching was delayed for 10 days starting from P35 with bumetanide treatment, which is similar to ocular dominance plasticity (Figures 1, 3).

### No Impairment of Visual Acuity and Depth Perception in Bum Mice

**FIGURE 4:**
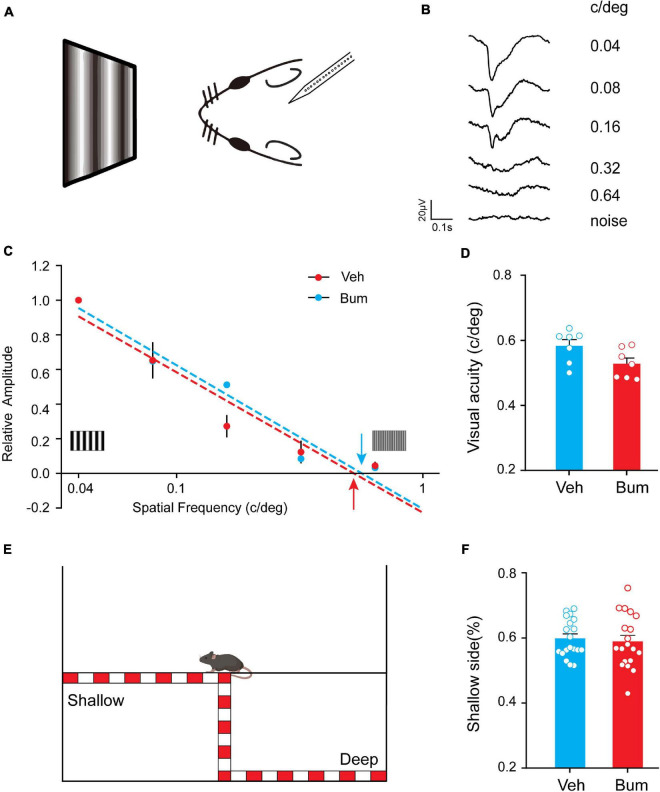
Bum mice displayed normal visual acuity and depth perception. **(A)** Experimental paradigm. With increasing spatial frequencies, visual evoked potential (VEP) decrease in amplitude and increase in latency. At.64 c/deg spatial frequency, VEPs are barely distinguishable from a response to a blank field (noise). **(B)** Representative example of VEPs in response to gratings of different spatial frequency (numbers to the right of tracings). **(C)** VEP amplitudes normalized to the mean amplitude at 0.04 c/deg; dashed lines are linear fits to the data, respectively. Estimated visual acuities (red and blue) are taken as the extrapolation to 0 level of the fitting lines for Bum (red) and Veh (blue) mice at P35. **(D)** Summary of visual acuity in two groups. Visual acuity of Bum animals (0.52 ± 0.016 c/deg, seven mice) is similar to Veh mice (0.58 ± 0.018 c/deg, seven mice, *p* = 0.053, unpaired *t*-test). **(E)** Schematic diagram of the visual cliff tests. **(F)** No difference was detected in the time fraction on the shallow side of arena between Bum (0.60 ± 0.013; mice = 19) and Veh (0.59 ± 0.018; mice = 19) animals at P35 (unpaired *t*-test, *p* = 0.68). Each circle indicates individual mouse. Data were expressed as mean ± SEM.

We then asked whether the transient disruption of binocular matching for orientation preference in Bum mice could affect the visual perceptive abilities of the animal. We performed an electrophysiological estimate of visual acuity (Figures 4A,B, see section “Materials and Methods”) between Bum and Veh animals at P35. We found that visual acuity of Bum animals (0.52 ± 0.016 c/deg, seven mice) is slightly but not significantly lower than Veh mice (0.58 ± 0.018 c/deg, seven mice, *p* = 0.053, Figures 4C,D). Visual cliff was used to assess stereoscopic vision in mice (Figure 4E). Previous studies showed that mice with normal binocular vision spent a longer time on the shallow side, and this difference was lost following MD, suggesting deficits in differentiating the shallow and deep sides (Gianfranceschi et al., 2003; Baroncelli et al., 2013). The whiskers were trimmed before the task to minimize somatosensory inputs. If mice can visually discriminate a drop-off at the edge of the cliff, they will prefer to explore the safe side. The behavioral performance of mice was measured by the percentage of time they spent on the safe side of the box. Our results showed that time spent on the shallow side was comparable between the Bum and Veh mice (*p* = 0.68). The fact that visual cliff test did not reflect the mismatching effect of Bum could be due to that it is not sensitive enough to detect the small and unstable orientation difference between the two eyes in Bum mice at P35. Overall, these results suggested that bumetanide treatment may specifically disrupt the development of binocular matching in visual cortical neurons without affecting basal visual properties such as visual acuity and stereopsis.

**FIGURE 5:**
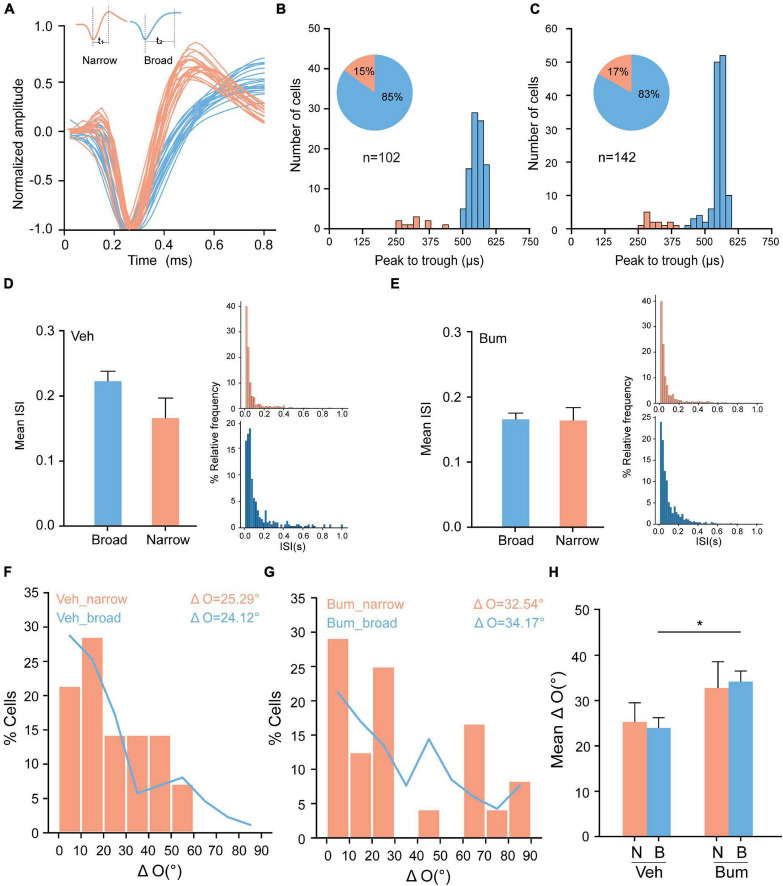
Bum treatment causes binocular mismatching of broad neurons. **(A)** Isolated units were classified as excitatory or inhibitory based on characteristics of their waveform. Example trace (20 spikes) from two units, one classified as putative inhibitory (orange) and one as putative excitatory (blue). **(B,C)** Distribution of peak to trough time of spike waveforms in Veh and Bum mice. In total, ∼15% of the units in both groups were classified as putative inhibitory (15%, 15 of 122; 17%, 24 of 142 units from Veh and Bum animals, respectively). **(D)** Left, mean inter-spike interval (ISI) of broad and narrow neurons for Veh mice at P35. Right, the ISI distribution for narrow (top) and broad (bottom) unit sample, the data were binned at 20 ms from 0 to 1 s. **(E)** The same as for **(D)** in Bum mice at P35. **(F,G)** Distribution of interocular difference of preferred orientation (“ΔO”) in both narrow and broad neurons of Veh and Bum groups: **(F)** Veh mice (narrow: ΔO = 25.29 ± 4.07°, 15 cells, nine mice; broad: ΔO = 24.26 ± 2.21°, 87 cells). **(G)** Bum mice (narrow: ΔO = 32.54 ± 5.7°, 24 cells, 14 mice; broad: ΔO = 34.17 ± 2.32°, 118 cells). **(H)** Mean ΔO of narrow and broad neurons in both Veh and Bum animals (*p* = 0.0151, Veh_broad vs. Bum_broad; *p* = 0.772, Veh_narrow vs. Bum_narrow; One-way ANOVA with Tukey’s *post hoc* test). N: Narrow, clear orange: Broad, pale blue). Data were expressed as mean ± SEM. *p < 0.05.

During the functional maturation of the mouse visual cortex, the miR-132/212 family is one of the most up-regulated miRNAs. The deletion of miR-132/212 does not affect the development of narrow-spiking units, suggesting that the disruption of binocular matching in miR-132/212 KO mice was not due to an alteration of narrow-spiking inhibitory neurons, but exclusively in broad-spiking excitatory neurons (Mazziotti et al., 2017). To further explore which type of neuron causes binocular mismatching in the specific time window in Bum mice, we classified neuronal units into two different classes: narrow-spiking (putative inhibitory) and broad-spiking (putative excitatory) units on the basis of their spike waveforms (Niell and Stryker, 2008; Figure 5A, see section “Materials and Methods” for details). Our results revealed that ∼15% of the units in both groups were classified as putative inhibitory (15%, 15 of 122; 17%, 24 of 142 units) from Veh and Bum animals, respectively (Figures 5B,C). To assure this classification could really distinguish the normal and fast-spiking neurons, we investigated the inter-spike interval (ISI) of broad and narrow units. We found that the mean ISI of narrow units (0.17 ± 0.029) is slightly less than broad units (0.22 ± 0.015, *p* = 0.13) in Veh mice (Figure 5D), whereas mean ISI were similar between the two types in Bum (broad: 0.17 ± 0.009; narrow: 0.16 ± 0.019, *p* = 0.90, Figure 5E). These data suggested that in Veh mice, narrow-spiking neurons did show higher spiking probabilities than broad-spiking neurons, while bumetanide might specifically facilitate the spiking activity of broad-spiking neurons. Furthermore, across the population, ΔO in broad-spiking neurons of Bum mice was significantly greater than age-matched Veh mice (Bum: ΔO = 34.17 ± 2.32°, 118 cells; Veh: ΔO = 24.26 ± 2.21°, 87 cells, *p* = 0.0154). While ΔO in narrow-spiking neurons of Bum mice was only partially greater than Veh mice (Bum: ΔO = 32.54 ± 5.7°, 24 cells; Veh: ΔO = 25.29 ± 4.07°, 15 cells, *p* = 0.772, one-way ANOVA and Tukey’s *post hoc* test, Figures 5F–H). Taken together, our results indicate that in Bum mice, a certain degree of mismatching occurred in the broad neurons, which covered the majority of total cortical neurons, played an important role in the transient binocular mismatching, probably through an enhancement in their excitability.

### Binocular Mismatching Was Accompanied by Changes in Excitatory Synaptic Structural and Molecular Properties at P35 With Bumetanide Treatment

**FIGURE 6:**
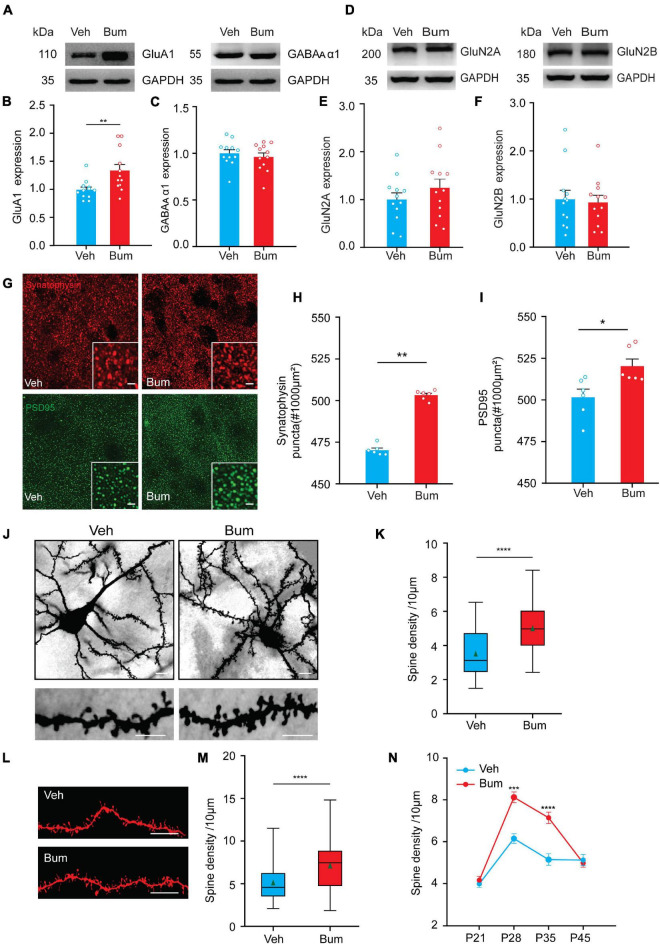
Binocular mismatching of bumetanide treatment is accompanied by changes in neuronal synaptic properties. **(A)** Cropped images of immunoblotting for GluA1 and GABA_A_ α1 on protein extracts from P35 V1 of Veh and Bum mice. **(B,C)** Quantification of GluA1 and GABA_ A_ α1 expression levels in Bum and Veh mice (GluA1: *p* = 0.0036; GABA_A_ α1: *p* = 0.78, 12 mice per group; Mann-Whitney rank-sum test). **(D)** Cropped images of immunoblotting for GluN2A and GluN2B on protein extracts from P35 V1of two groups. **(E,F)** Quantification of GluN2A and GluN2B expression levels in Bum and Veh mice (GluN2A: *p* = 0.48; GluN2B: *p* = 0.97, 12 mice per group; Mann-Whitney rank-sum test). GAPDH was used as the internal standard. Each circle indicates individual mouse. **(G)** Confocal images (60×, single plane) show synaptophysin (SYN) (red) and postsynaptic PSD-95 (green) proteins in V1b from Bum and Veh mice at P35. Insets in the lower right corner of the images are 2 × zoom-in views of SYN  and PSD-95  puncta. Scale bar, 5 μm. **(H,I)** Quantification of synaptophysin and PSD-95 positive puncta in the Bum compared to Veh animals (SYN: *p* = 0.0022; PSD-95: *p* = 0.015; Mann-Whitney rank-sum test), six mice per group, three sections per mouse, and four images per section. Each circle represents the average protein density of all brain slices of a mouse. **(J)** Upper panel, representative images of layer V/VI pyramidal neurons in V1 of Bum and Veh brain sections at P35 with Golgi staining. Scale bar, 20 μm. Lower panel, representative images of a dendritic branch in Veh and Bum mice. Scale bar, 10 μm. **(K)** Quantification of the dendrite spine density in V1 of Veh (3.52/10 μm, 41 dendrites, four mice) and Bum (5.01/10 μm, 31 dendrites, three mice) animals at P35 (*p* < 0.0001; Mann-Whitney rank-sum test). **(L)** Representative images of DiI dye staining of lateral branches of dendrites at P35 in Veh and Bum mice. Scale bar, 10 μm. **(M)** The same as **(K)**, quantification of spine density in Bum and Veh mice (Bum: 7.14/10 μm, 116 dendrites, five mice; Veh: 5.14/10 μm, 58 dendrites, four mice, *p* < 0.0001; Mann-Whitney rank-sum test). **(N)** Average spine density in Bum and Veh visual cortical neurons at different ages. No difference at P21 (Bum: 4.98/10 μm, 71 dendrites, five mice; Veh: 4.07/10 μm, 86 dendrites, four mice) and P45 (Bum: 4.98/10 μm, 63 dendrites, three mice; Veh: 4.07/10 μm, 108 dendrites, five mice); the density of dendritic spines in Bum animals were significantly higher than Veh littermates at P28 and P35 (P28: Bum, 8.12/10 μm, 72 dendrites, four mice; Veh, 6.15/10 μm, 83 dendrites, five mice; P28: *p* = 0.0004; P35: *p* < 0.0001; Two-way ANOVA with Tukey’s post hoc test). For **(K,M)** data are represented as box charts. For each box chart, the central horizontal line represents the median value, the other two horizontal lines are the 25th and 75th percentiles, and error bars denote the 5th and 95th percentiles. The green triangle symbol indicates the average value. Data were expressed as mean ± SEM except for box charts. *p < 0.05, **p < 0.01, ***p < 0.001, ****p < 0.0001.

The above results showed that bumetanide treatment prolonged the normal critical period for 10 days (Figure 1). Thus, we hypothesize that the increase in synaptic plasticity induced by bumetanide treatment leads to increased neuronal instability, causing the postponed binocular matching of orientation preference. Our results have revealed that the binocular mismatching of Bum mice might mainly be caused by excitatory neurons (Figure 5). Changes in the balance of excitation and inhibition (E/I balance) are thought to regulate the initiation and termination of the critical period for OD plasticity (Baroncelli et al., 2010; Hensch and Quinlan, 2018). During the critical period, the inhibitory transmission gradually enhances its driving force (Hensch et al., 1998; Sadahiro et al., 2020), which eventually lower the system plasticity. We thus examined the relative protein expression levels of GABAergic/Glutamatergic receptors with bumetanide treatment. GluA1 subunit of AMPA receptor is necessary for ocular dominance plasticity during the critical period (Ranson et al., 2013), and we found that bumetanide treatment resulted in increased expression of GluA1 compared to the Veh control at P35 (Figures 6A,B, *p* = 0.0036). The α1 subunit containing GABA_A_ receptor which is localized on the soma of pyramidal neurons receiving inhibitory synaptic inputs from PV interneurons increased its expression during development, which further implicates its control of the critical period (Hensch, 2005). However, we found no difference in GABA_A_ α1 protein expression in cortical neurons between two groups at P35 (Figures 6A,C, *p* = 0.9352). Furthermore, there is evidence suggesting that NMDA receptor is closely linked to the plasticity of excitatory and inhibitory synapses, and it may be necessary for experience-dependent visually evoked responses during the critical period (Daw et al., 1999; Quinlan et al., 1999; Sawtell et al., 2003; Sato and Stryker, 2008; Kanold et al., 2009). GluN2B is the predominant subunit in juvenile visual cortex, which is gradually replaced by GluN2A subunit during the maturation. However, in Bum mice, GluN2B and GluN2A expression levels were indistinguishable from those observed in Veh mice at P35 (Figures 6D–F, GluN2A: *p* = 0.48; GluN2B: *p* = 0.97). However, what we observed in the protein expression pattern could be undermined due to the fact that glia were also included in the brain tissue.

Stable pre to post-synaptic connections and mature dendritic spine architecture are necessary structural elements for proper functional encoding and expression (Leitner et al., 2013; Harwell and Coleman, 2016; Chakroborty et al., 2019). Thus, we further investigated whether bumetanide treatment produced any alteration in the expression of presynaptic and postsynaptic molecules in V1b. The expression of the global presynaptic marker synaptophysin (SYN) and excitatory postsynaptic marker (PSD-95) were quantified by fluorescent labeling (Figures 6G–I). We found a significant increase in the number of SYN and PSD-95 puncta in Bum mice, respectively, compared to age-matched Veh mice (SYN: *p* = 0.0022; PSD-95: *p* = 0.015), which suggested an increase in the synaptic density, especially the excitatory synapses of the visual cortex in Bum mice.

As dendritic spines are known to play a significant role in neuronal plasticity and synaptic integration through structural rearrangement of the excitatory synapses during development (Ultanir et al., 2007), we analyzed the spine density of apical and basal dendrites of layer V/VI pyramidal neurons in V1 using the rapid Golgi impregnation method (Das et al., 2013; Ka et al., 2016; Du, 2019) in Bum and Veh mice at P35. We revealed that spine density of Bum mice (5.01/10 μm) was significantly higher compared to Veh littermates (3.52/10 μm) at P35 (Figures 6J,K, *p* < 0.0001). This result was confirmed with DiI assay in Veh and Bum mice. DiI is distributed and diffused through the cell membrane to fully highlight the dendrites and spinous processes, thereby providing a clear outline for neuronal processes (Ultanir et al., 2007; Cheng et al., 2014). Our results showed that the spine density in apical and basal dendrites of layer V/VI pyramidal neurons in Bum animals (7.14/10 μm) was significantly higher than Veh mice at P35 (5.14/10 μm, *p* < 0.0001; Figures 6L,M). Furthermore, we examined the spine density of the two groups of mice at other ages by DiI staining. Indeed, spine density increased similarly from P21 (Veh: 4.07/10 μm; Bum: 4.98/10 μm) and was maximal at P28 in both Bum (8.12/10 μm) and Veh mice (6.15/10 μm; Figure 6N, two-way ANOVA with Tukey’s *post hoc* test, *p* < 0.001). Bumetanide treatment enhanced the spine density but had no effect on binocular matching at P28, probably due to the fact that for Veh at P28, the plasticity is high enough, and an even higher spine density could not further change the binocular matching. There was no difference of spine density between Bum (4.98/10 μm) and Veh (4.07/10 μm) animals at P45. The density of spines in the Veh group at P35 (5.14/10 μm) and P45 was similar, indicating that synaptic plasticity in the control group had reached adult status at P35. These results suggested that binocular mismatching in Bum mice at P35 (7.14/10 μm) may be due to a heightened plasticity caused by molecular and structural modifications at the excitatory synapses.

### Diazepam/CPP Reversed Both the Extended Ocular Dominance Plasticity and the Binocular Mismatching in Bum Mice at P35

**FIGURE 7:**
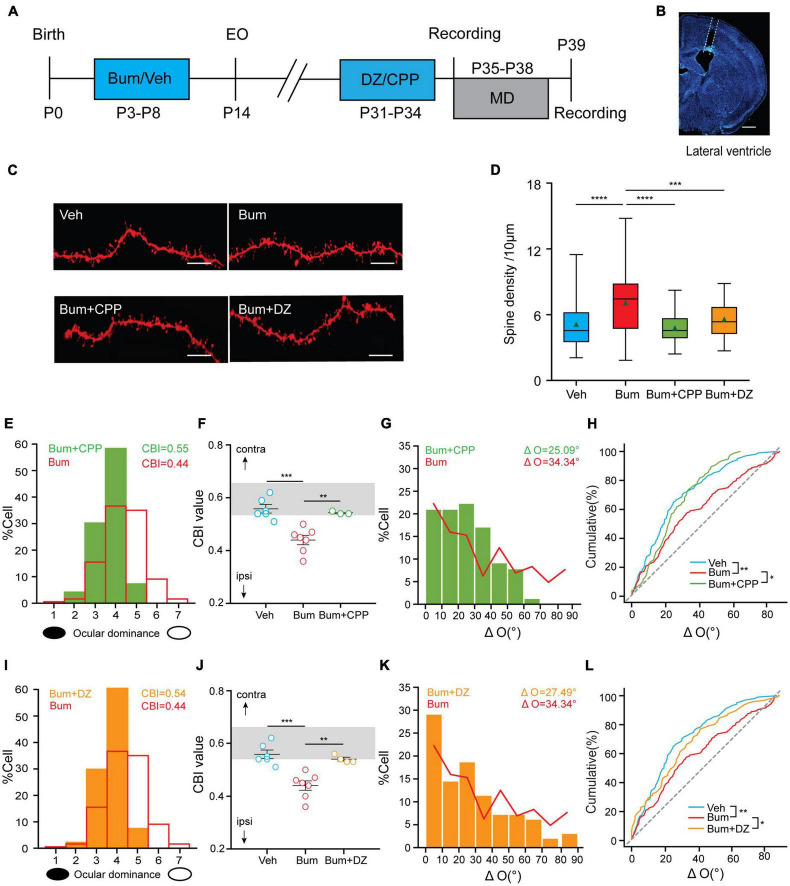
Diazepam/[(R, S)-3-(2-carboxypiperazin-4-yl) propyl-1-phosphonic acid (CPP)] restores the extended OD plasticity and rescues the mismatching deficit in Bum mice. **(A)** Schematic of experimental design. **(B)** Image showing traces of cannula in lateral ventricle, scale bar, 500 μm. **(C)** Representative images of DiI dye staining of lateral branches of apical dendrites at P35 in Veh and Bum, Bum + CPP and Bum + diazepam (DZ) mice. Scale bar, 10 μm. **(D)** Spine density at P35 Bum (7.14/10 μm) was significantly higher than age-matched Veh (5.14/10 μm, 58 dendrites, four mice), Bum + CPP (4.81/10 μm, 56 dendrites, four mice), and Bum + DZ (5.6/10 μm, 49 dendrites, four mice). The data are summarized by a box chart, and the horizontal lines in the box denote the 25th, 50th, and 75th percentile values. The error bars denote the 5th and 95th percentile values. The green triangle symbol indicates the average value. *p* = 0.0006, Bum vs. Bum + DZ; *p* < 0.0001, Veh vs.Bum; *p* < 0.0001, Bum vs. Bum + CPP; *p* = 0.28, Bum + CPP vs. Bum + DZ. **(E,F)** Administration of CPP restores the extended OD plasticity. **(E)** CPP blocked the OD shift in Bum mice at P35 (green histogram). The red solid line represents the OD distribution of Bum mice with 4 days MD. **(F)** OD plasticity was quantified by the values of CBI (Veh: 0.56 ± 0.015, six mice; Bum: 0.44 ± 0.016, seven mice; Bum + CPP: 0.55 ± 0.027, three mice; *p* = 0.0002, Veh vs. Bum; *p* = 0.0066, Bum vs. Bum + CPP; open circles represent individual CBIs for each animal, One-way ANOVA with Tukey’s *post hoc* test). The gray box indicates the typical range of CBI values for non-deprived mice. **(G)** Distribution of the difference in orientation preference in Bum + CPP (ΔO = 25.09 ± 1.83°, 75 cells, five mice). The red solid curve represents the ΔO distribution of Bum (ΔO = 34.34 ± 2.15°, 142 cells, 14 mice). **(H)** Cumulative distribution of ΔO in Bum + CPP, Bum, and Veh (ΔO = 24.26 ± 1.54°, 102 cells, nine mice. *p* = 0.0035, Veh vs. Bum; *p* = 0.30, Veh vs. Bum + CPP; *p* = 0.046, Bum + CPP vs. Bum, K-S test). **(I,J)** the same as **(C,D)** administration of DZ restores the extended OD plasticity in Bum mice (Bum + DZ: 0.54 ± 0.006, four mice; *p* = 0.0002 Veh vs. Bum; *p* = 0.0031 Bum vs. Bum + DZ). **(K)** The same as **(E)** in Bum + DZ (ΔO = 27.49 ± 2.30°, 94 cells, five mice). **(L)** The same as **(F)** in Bum + DZ, Bum, and Veh groups (*p* = 0.42, Veh vs. Bum + DZ; *p < 0.05, **p < 0.01, ***p < 0.001, ****p < 0.0001, Bum + DZ vs. Bum). P35 for binocular matching; P35 + 4d MD for ocular dominance plasticity. Data were expressed as mean ± SEM except for box charts.

The above experiments revealed that binocular matching was disrupted and accompanied by changes in neuronal synaptic structural properties and increased GluA1 expression in Bum mice at P35. To test the cause-effect relationship between binocular matching and E/I balance, we examined whether the transient binocular mismatching could be rescued by increasing inhibition or decreasing excitation. To block the excitatory synaptic transmission through NMDA receptors (Wang et al., 2010), we intraperitoneally injected NMDA receptor antagonist CPP (Sato and Stryker, 2008) or lateral ventricle (*i.c.v.*) microperfusion of GABA receptor agonist DZ via cannula (Baroncelli et al., 2010) into Bum mice starting from P31(Bum + CPP or Bum + DZ), with continuous injections every ∼24 h for 4 days (Figures 7A,B, see section “Materials and Methods”), and compared them to the Bum and Veh data we obtained in the previous sections. We first investigated the changes of dendritic spine density in Bum + CPP and Bum + DZ mice at P35 by DiI staining. We found that the dendritic spine density of the Bum + CPP (4.81/10 μm) and Bum + DZ (5.6/10 μm) were comparable to Veh mice (5.14/10 μm), and significantly lower than Bum group (7.14/10 μm, one-way ANOVA with Tukey’s *post hoc* test; Figures 7C,D), suggesting that CPP and DZ could reverse the spine density to normal level in Bum mice.

What effects will these two pharmacological manipulations have on ocular dominance and binocular matching in Bum mice? After 4d MD starting at P35, CBI from individual animals in Bum + CPP mice (0.54 ± 0.003, 96 cells, three mice) were prominently higher than those in Bum mice (0.44 ± 0.016, 354 cells, seven mice), which was similar to Veh mice with MD (0.56 ± 0.014, 463 cells, six mice; Figures 7E,F). These data suggested that the ocular dominance plasticity that is prolonged by Bum has similar dependence on NMDA receptors as during the normal development. Next, we further investigated the effect of CPP on binocular matching in Bum mice. Distribution of ΔO of Bum + CPP mice (ΔO = 25.09 ± 1.83°, 79 cells, seven mice) was similar to age-matched Veh (ΔO = 24.26 ± 1.97°, 102 cells, nine mice) and both were significantly lower than Bum mice (ΔO = 34.34 ± 2.14°, 142 cells, 14 mice; Figures 7G,H). Therefore, the maturation of binocular matching during the prolonged critical period is also dependent on NMDA receptors, similar to the normal critical period (Wang et al., 2010). We also evaluated ocular dominance plasticity in Bum animals treated with DZ (Bum + DZ) with 4d MD by recording the responses of single-unit in the V1b contralateral to the deprived eye. DZ administration completely prevented the ocular dominance shift observed in Bum mice at P35 (Bum + DZ: 0.54 ± 0.0061, 134 cells, four mice; Bum: 0.44 ± 0.016, 142 cells, seven mice), and we found no differences of CBI between Bum + DZ and Veh group (0.56 ± 0.014, 102 cells, nine mice). Our results indicated that the increased visual cortical inhibition by DZ could reverse the enhanced plasticity of Bum at P35 (Figures 7I,J). Furthermore, ΔO in Bum + DZ mice (27.49 ± 2.3°, 96 cells, six mice) was similar to age-matched Veh controls, yet significantly lower than Bum animals, indicating that increasing inhibition in visual cortical neurons by DZ in Bum mice restores binocular matching of orientation preference at P35 (Figures 7K,L). These data, together with the above morphological results, indicated that the manipulations concerning the E/I balance could rescue the binocular mismatching in Bum mice at P35.

### DHF Inhibits Ocular Dominance Plasticity but Does Not Rescue the Binocular Matching in Bum Mice at P35

**FIGURE 8:**
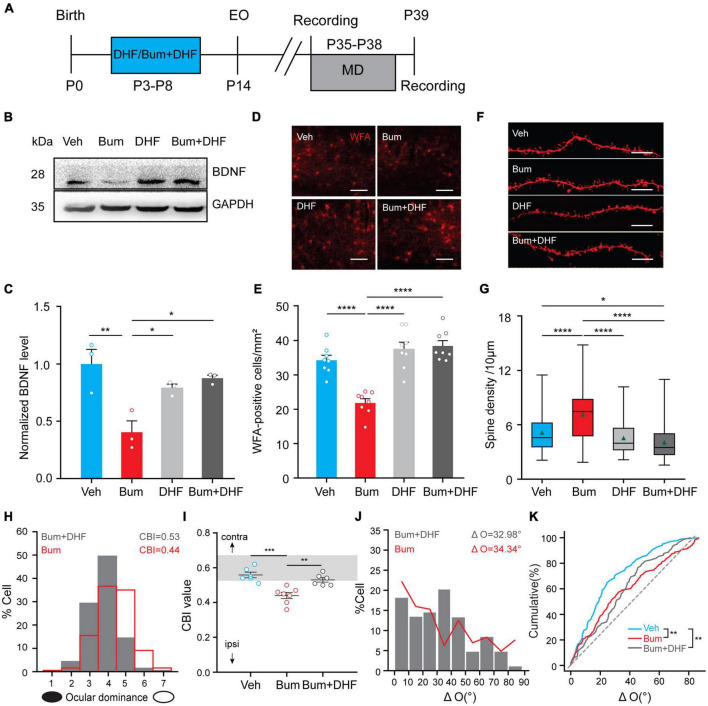
Dihydroxyflavone (DHF) inhibits OD plasticity but does not rescue the binocular matching in Bum mice at P35. **(A)** Schematic graphic of the experimental protocol. **(B,C)** Cropped images and quantifications of immunoblotting for BDNF on protein extracts from P35 visual cortices of four groups (Veh, Bum, Bum + DHF, DHF). GAPDH was used as the internal standard (*p* = 0.0045, Veh vs. Bum; *p* = 0.0171, Bum vs. DHF; *p* = 0.047, Bum vs. Bum + DHF, three mice per group; Mann-Whitney rank-sum test). Each circle indicates individual mouse. **(D)** Cropped images of WFA-stained perineuronal nets (red) in V1b at P35 in four groups (Veh, Bum, DHF and Bum + DHF)Scale bar, 200 μm. **(E)** Quantification of density of WFA-positive cells in four groups. The density of positive cells in Bum mice is statistically lower than other groups (*p* < 0.0001, eight mice per group, five sections per mouse, two mages per section; One-way ANOVA with Tukey’s *post hoc* test). **(F)** Representative images of DiI dye staining of lateral branches of apical dendrites at P35 in Veh and Bum, Bum + DHF and DHF mice. Scale bar, 10 μm. **(G)** Spine density at P35 Bum (7.14/10 μm) was significantly higher than age-matched Veh (5.14/10 μm, 58 dendrites, four mice), Bum + DHF (4.05/10 μm, 111 dendrites, five mice), DHF (4.53/10 μm, 68 dendrites, four mice). The data are summarized by a box chart, and the horizontal lines in the box denote the 25th, 50th, and 75th percentile values. The error bars denote the 5th and 95th percentile values. The green triangle symbol indicates the average value. *p* = 0.0169, Veh vs. Bum + DHF; *p* < 0.0001, Veh vs. Bum; *p* < 0.0001, Bum vs. DHF; *p* < 0.0001, Bum vs. Bum + DHF. **(H)** DHF co-treatment with bumetanide (Bum + DHF) blocked the OD shift in Bum mice. The red solid line represents the OD distribution of Bum mice with 4 days MD. **(I)** OD plasticity quantified by the values of CBI (Veh: 0.56 ± 0.015, six mice; Bum + DHF: 0.53 ± 0.012, six mice; Bum: 0.44 ± 0.016, seven mice. *p* = 0.0002, Veh vs. Bum; *p* = 0.0028, Bum vs. Bum + DHF, One-way ANOVA with Tukey’s *post hoc* test). Open circles represent individual CBIs for each animal. **(J)** Distribution of the difference in orientation preference in Bum + DHF (ΔO = 32.98 ± 2.35°, 82 cells, 16 mice) and Bum (ΔO = 34.34 ± 2.15°, 142 cells, 14 mice) groups. **(K)** Cumulative distribution of ΔO in Bum + DHF, Bum, and Veh (ΔO = 24.26 ± 1.54°, 102 cells, nine mice; *p* = 0.0035, Veh vs. Bum; *p* = 0.0031, Veh vs. Bum + DHF; *p* = 0.929, Bum + DHF vs. Bum, K-S test). P35 for binocular matching; P35 + 4d MD for ocular dominance plasticity. DHF, light gray; Bum + DHF, dark gray. Data were expressed as mean ± SEM except for box charts. *p < 0.05, **p < 0.01, ***p < 0.001, ****p < 0.0001.

Interestingly, it has been previously reported that early 7,8-dihydroxyflavone DHF (BDNF mimetic drug) co-treatment with bumetanide could rescue the extended OD plasticity, probably through accelerating the maturation of perineuronal nets (PNNs) in rat V1 at P35 (Deidda et al., 2015). Early interference with depolarizing GABA by bumetanide treatment impairs both the synthesis and release of BDNF. In our experiments, we did replicate these effects of DHF in Bum mice. Treatment of DHF together with bumetanide (Bum + DHF, Figure 8A) also rescued BDNF expression (Figures 8B,C) and PNNs (Figures 8D,E). We also examined the changes of dendritic spine density in DHF and Bum + DHF mice at P35 by DiI staining. We found that the dendritic spine density of the DHF (4.53/10 μm) was comparable to Veh mice (5.14/10 μm), and significantly lower than Bum group (7.14/10 μm, one-way ANOVA with Tukey’s *post hoc* test; Figures 8F,G), suggesting that DHF *per se* could not affect the spine density. Interestingly, the co-treatment of DHF and Bum (4.05/10 μm) reduced the density of dendritic spines to a certain extent compared to the control group (Figures 8F,G). After 4d MD starting at P35, CBI from individual animals in Bum + DHF mice (0.53 ± 0.012, 208 cells, six mice) were prominently higher than those in Bum mice (0.44 ± 0.016, 354 cells, seven mice), which was similar to Veh mice with MD (0.56 ± 0.014, 463 cells, six mice; Figures 8H,I). This result indicates that the co-treatment of Bum and DHF block OD plasticity in Bum mice, which is consistent with previous studies in rats (Deidda et al., 2015). Although cumulative distribution of ΔO showed that Bum + DHF mice had a significant larger ΔO than Veh mice, which is indistinguishable from Bum mice (Veh: ΔO = 24.26 ± 1.97°, nine mice, 102 cells; Bum: ΔO = 34.34 ± 2.14°, 14 mice, 142 cells; Bum + DHF: ΔO = 32.98 ± 2.35°, 15 mice, 82 cells; Figures 8J,K). There is no significant difference in ΔO between the Bum + DHF and Bum mice. The results that DHF failed to rescue the binocular matching at P35 in Bum mice, combining with the above DZ/CPP data, suggested different mechanisms for the rescue of extension of ocular dominance plasticity and binocular matching.

### Experiencing Normal Vision During the Extended Critical Period Alone Is Sufficient for the Maturation of Binocular Matching Following LTMD in Bum Mice

**FIGURE 9:**
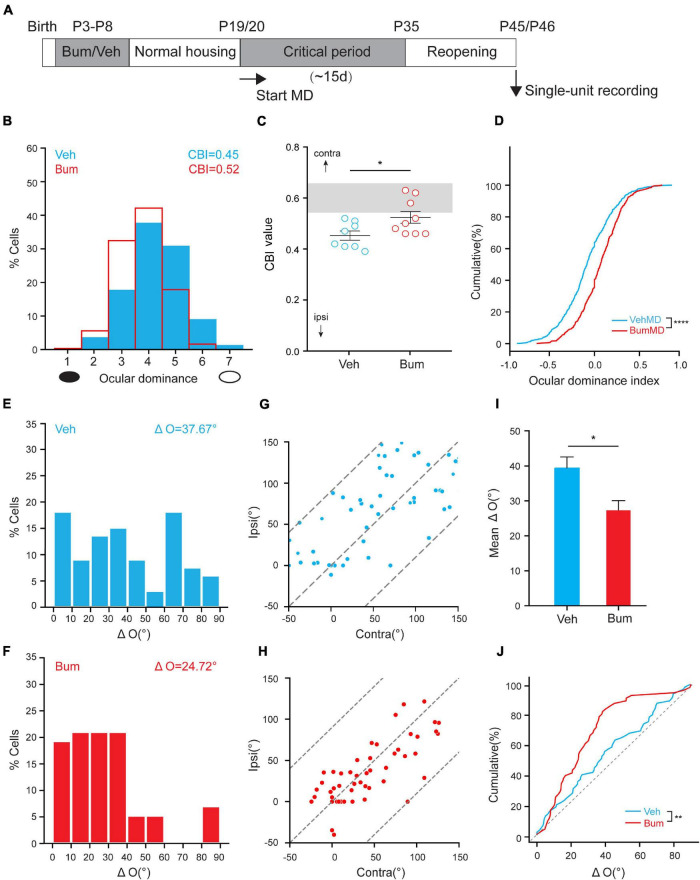
The extended critical period with normal vision rescued binocular matching following long-term monocular deprivation (LTMD) in Bum mice. **(A)** Schematic of experimental design. **(B)** OD distribution for Veh and Bum mice with LTMD. The OD distribution was shifted toward the non-deprived eye for Veh mice, while Bum mice showed contralateral bias (*p* = 0.031, Mann-Whitney rank-sum test). **(C)** OD plasticity was quantified by the values of CBI (Veh: 0.45 ± 0.016, eight mice; Bum: 0.52 ± 0.021, nine mice). Open circles represent individual CBIs for each animal. **(D)** OD cumulative distribution for Veh and Bum group. OD distribution for Bum group (610 cells, nine mice) was statistically different from Veh mice (686 cells, eight mice, *p* < 0.0001, K-S test). **(E,F)** Distribution of the difference in interocular orientation preference in Veh and Bum mice. **(E)** Veh ΔO = 37.67 ± 3.24°; **(F)** Bum ΔO = 24.72 ± 2.82°. **(G,H)** Correlation of monocular orientation preference in Veh and Bum mice. **(G)** Veh: *r* = 0.66, *p* < 0.0001, 69 cells, eight mice; **(H)** Bum: *r* = 0.72, *p* < 0.0001, 60 cells, nine mice. The dotted lines bound the region in which the data points can lie. **(I)** Mean ΔO was significantly higher in Veh than Bum animals (*p* = 0.0108, Mann-Whitney rank-sum test). **(J)** Cumulative distribution of ΔO for Bum and Veh mice (*p* = 0.0044, K-S test). Data were expressed as mean ± SEM. *p < 0.05, **p < 0.01, ****p < 0.0001.

It has been previously reported that long-term MD (LTMD) spanning the entire critical period could induce a life-long mismatching of orientation preference, which could not be spontaneously rescued even with the reopening of the deprived eye in adulthood, mimicking amblyopic patients (Wang et al., 2010; Levine et al., 2017). To further investigate the effect of the extended critical period on binocular matching, we examined whether binocular matching could be completed merely by the restoration of normal vision during the 10d extended critical period following LTMD in Bum mice. Both Veh/Bum mice were monocular deprived from P19/P20 (onset of the critical period) until P35 (closure of the normal critical period), and returned to home cage for ∼10 days after reopening the deprived eye. Single-unit recording was performed at P45/46 acro*s*s all cortical layers in the binocular zone of V1 contralateral to the deprived eye and the monocular orientation tuning of each unit was assessed using drifting sinusoidal gratings (Figure 9A). As shown in Figures 9B–D, the OD distribution of the animals subjected to normal visual experience after LTMD showed significant shift toward the ipsilateral eye (open eye) in Veh animals, while in Bum the contralateral bias was recovered merely by the 10-day normal binocular vision during P35-P45. Interestingly, we found that smaller ΔO was seen in Bum mice after the extended critical period with restored binocular visual experience (ΔO = 24.72 ± 2.82°, 60 cells, nine mice, Figure 9F). In contrast, in Veh mice, most cells had ΔO higher than 20°, with a mean of 37.67 ± 3.24° (69 cells, eight mice, *p* = 0.0108, Figure 9E), similar to previous findings (Wang et al., 2013; Levine et al., 2017). Such similarity in the preferred orientations between the two eyes was observed across the population in Bum mice (correlation coefficient *r* = 0.72, Figure 9H), which was higher than Veh mice (*r* = 0.66, Figure 9G). Overall, more cells in Veh mice had greater ΔO than Bum mice. Cumulative distribution of ΔO also showed that Veh mice had a significant larger ΔO than Bum mice (*p* = 0.0044, K-S test; Figures 9I,J). Our results indicated that the extended critical period with normal vision is sufficient for the complete re-matching of the mismatched orientation preference in amblyopic Bum mice.

### NgR1^–/–^ Mice Showed Impaired Binocular Matching and Depth Perception in Adulthood

**FIGURE 10:**
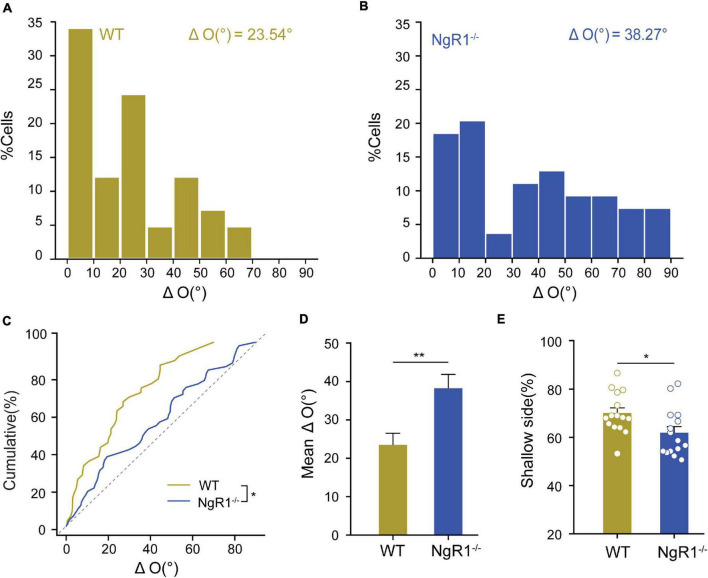
NgR1^–/–^ disrupts normal maturation of binocular matching and depth perception. **(A,B)** Distribution of interocular difference of preferred orientation (“ΔO”) in wild-type (WT) and NgR1^–/–^ groups: **(A)** WT (ΔO = 23.53 ± 2.97°, 41 cells, five mice); **(B)** NgR1^–/–^ mice (ΔO = 38.27 ± 3.59°, 54 cells, six mice). **(C)** Cumulative distribution of ΔO for Veh and NgR1^–/–^ groups (*p* = 0.020, K-S test). **(D)** Mean ΔO for two groups (*p* = 0.0094, Mann-Whitney rank-sum test). **(E)** WT animals spent a higher percentage of time on the safe side with respect to NgR1^–/–^ mice (15 mice per group, *p* = 0.032, unpaired *t*-test). Each circle indicates individual mouse. Data were expressed as mean ± SEM. *p < 0.05, **p < 0.01.

The above results indicate that early bumetanide treatment caused a transient binocular mismatching in a specific time window. It remains unknown if an even longer critical period into adulthood could have similar effects. In order to further investigate the role of the critical period closure on the binocular matching process, we performed *in vivo* extracellular single-unit recordings of V1 neuron from both P60-P90 WT and Nogo-66 receptor knockout mice (NgR1^–/–^). Nogo receptor is a myelination-related membrane receptor, which exists widely on both neurons and glia. When bound with Nogo protein, it can form a complex with extracellular matrix, induce the myelination process, and inhibit axon bifurcation. Knocking out Nogo receptor can maintain the ocular dominance plasticity in V1 at a high level, which could extend the critical period into adulthood (McGee et al., 2005). To know if knocking out Nogo receptor affects the normal completion of binocular matching, we measured the orientation preference in V1 neurons of WT and NgR1^–/–^ mice in response to drifting gratings moving at 12 orientations, and compared the orientation preference between the contralateral and ipsilateral eye. By calculating the ΔO of orientation preference between the two eyes (Figures 10A,B), we found that, in NgR1^–/–^, there were more cells accumulating around higher ΔO compared to WT controls (WT: ΔO = 23.54 ± 2.97°, 41 cells, five mice; NgR1^–/–^: ΔO = 38.27 ± 3.59°, 54 cells, six mice, *p* = 0.0094). Cumulative distribution of ΔO also showed that NgR1^–/–^ mice had a significant larger ΔO than WT mice (*p* = 0.020, K-S test; Figures 10C,D). These data suggested that in NgR1^–/–^ mice, the normal binocular matching process was suppressed even in adulthood.

Since the binocular matching is considered to be the foundation of stereoscopic vision, thus lacking of binocular matching could result in deficits in the stereoscopic perception. To test the depth perceptive ability, we performed visual cliff tests in both WT and NgR1^–/–^ adult mice (Han et al., 2017). The mice were placed in the center of a box with a transparent bottom extending outside of the edge of the bench top, revealing an apparent cliff (see section “Materials and Methods”). We found that NgR1^–/–^ mice showed a significant decrease of the preference for the safe side compared to WT controls (WT: 70.1 ± 2.2%, NgR1^–/–^: 62 ± 2.6%, *p* = 0.032, Figure 10E), suggesting that knocking out NgR1 disrupted the normal depth perception. The results suggested that prolonging the critical period into adulthood can also suppress the normal completion of binocular matching, which could further suppress the depth perception and stereoscopic vision.

## Discussion

During early postnatal development, the binocular neurons in primary visual cortex (V1) undergo a profound maturation process called binocular integration (Hubel and Wiesel, 1962). Binocular matching of orientation preference, one representative form of binocular integration, has been found in multiple species from rodents to primates, including humans (Bridge and Cumming, 2001; Gu and Cang, 2016). The current study elaborated that short-term critical period prolonging could transiently prevent binocular matching, which is accompanied by changes in neuronal synaptic structural properties. The extended critical period with normal vision is sufficient for the complete re-matching of certain degree of mismatching that occurred in the broad neurons in amblyopic mice. In addition, permanently extending critical period into adulthood in NgR1^–/–^ mice (McGee et al., 2005) can also suppress the normal completion of binocular matching and depth perception.

In many studies, manipulations that affect the critical period timing may regulate the overall development of the visual system. For example, keeping animals in the dark rendered the animals an immature visual system, thereby reducing the visual acuity, which would lead to a prolonged window of susceptibility to MD. Manipulations that lead to the premature closure of critical period plasticity, such as EE, overexpression of IGF-1, or BDNF, can also lead to accelerated cortical development (Wang and Kriegstein, 2008; Wang et al., 2013). In BDNF-OE mice, the binocular matching process fails to reach adult level, presumably due to the precocious closure of cortical plasticity (Wang et al., 2013). In contrast, in Bum mice, binocular matching does reach the normal adult level at P45, probably due to reinstatement of the transient abnormal heightened plasticity. Actually, in these BDNF-OE mice, the critical period for ocular dominance plasticity was both precociously initiated and closed. In other words, the entire time window for critical period was shifted forward ahead of the time window for normal binocular matching, which may completely disrupt the maturation of binocular matching. Conversely, bumetanide treatment only affects the closure of critical period, while the onset of critical period remains intact. And the critical period close is only temporarily delayed for 10 days, so when the extended critical period is closed, the binocular matching would be spontaneously recovered. Indeed, the temporarily increased spine density by Bum was also spontaneously recovered at P45 (Figure 6N). The effect of Bumetanide is also quite different from the CPP administration during the critical period that completely inhibit the plasticity would prevent the process of binocular matching, which also cannot be spontaneously recovered (Wang et al., 2010). Unlike Bum mice, interestingly, NgR1^–/–^ mice permanently extended the critical period (McGee et al., 2005; Stephany et al., 2016) and showed a life-long deficit in binocular matching (Figure 10).

What is the possible mechanism linking the effect of critical period prolonging with the postponed binocular matching? It is well appreciated that the alternation of the balance between excitation and inhibition is regarded as the major regulator for visual plasticity and its reactivation in the development (He et al., 2006; Harauzov et al., 2010). In Bum mice, the heightened plasticity during the extended critical period may be due to an acute consequence of decreased cortical inhibition or increased excitation, or both. Changes in the properties of α-amino-3-hydroxy-5-methyl-4-isoxazole-propionicacid (AMPA), N-methyl-d-aspartate (NMDA), and γ-gamma-aminobutyric acid (GABA) receptors have been suggested to mediate synaptic transmission interactions to determine the characteristics of synaptic plasticity during the development in the visual cortex (Santucci and Raghavachari, 2008). Previous studies showed that dark rearing prior to MD can decrease the GluN2A/GluN2B ratio and promote ocular dominance plasticity (Tropea et al., 2009), that GluA1 subunit of the AMPA receptor is necessary for open eye potentiation during the critical period (Ranson et al., 2013), and that GABA_A_ α1-containing inhibitory circuits were found to drive cortical plasticity (Fagiolini et al., 2004). In this study, we did not observe significant alteration of total protein levels in GluN2A, GluN2B, and GABA_A_ α1 subunit in visual cortex between Bum and Veh mice, albeit the ratio of GluN2A/GluN2B expression increased slightly. In contrast, the expression of GluA1 was remarkably elevated by the bumetanide treatment, indicating the heightened plasticity may be specifically due to the upregulation of GluA1 (Figure 6A). However, this did not rule out the possible involvement of NMDA and GABA receptors because we didn’t specifically quantify the receptor density on the synaptic membrane or record the mini excitatory post-synaptic current (EPSC)/inhibitory post-synaptic current (IPSC) amplitude and frequency, which directly reflect the synaptic efficacy.

It is reported that direct or indirect reduction of GABAergic inhibition promotes the ocular dominance plasticity in adulthood (Li et al., 2018). The enhancement of GABAergic transmission through chronic DZ infusion can modulate the appearance of binocular input coincidence (Wang et al., 2013). In addition, NMDA receptor is required for synaptic plasticity and binocular matching during critical period of normal development (Wang et al., 2010). CPP was shown to block MD-induced ocular dominance shift in both juvenile and adult mice, but has no effect on normally reared animals. We thus attempted to rescue the heightened plasticity *in vivo* by briefly enhancing inhibition or reducing excitation in Bum animals. We administered GABA receptor agonist diazepam locally or NMDA receptor antagonists CPP systemically in Bum mice and found that the extended ocular dominance plasticity and deficits of binocular matching at P35 were both rescued. The possible reason is that the establishment of binocular matching is an active and experience-dependent developmental process, which is closely related to the precise plasticity at different time point of the critical period. Bumetanide treatment induced a heightened plasticity at P35, the time point for normal critical period close, so DZ and CPP could either enhance inhibition or reduce excitation, both of which can reinstate the plasticity during the extended critical period, and rescue the deficits in binocular matching. Interestingly, bumetanide treatment did not affect GluN2A and GluN2B expression, indicating that the effect of CPP is to reduce the total excitation, which may not involve other signaling pathway mediated by specific NMDA subunits. Although we did not modulate AMPA receptors in this study, we hypothesize that reducing excitation through AMPA receptor blocker could show similar effects.

Early bumetanide treatment reduced the expression of BDNF, therefore, to examine if increasing the BDNF signaling could restore the binocular matching, 7,8-dihydroxyflavone (DHF), an analog of BDNF which can act as a powerful TrkB agonist (Jang et al., 2010), were administrated together with bumetanide postnatally. Our data showed that DHF could restore the normal closure of the critical period, accompanied by recovery of the normal expression of PNNs and BDNF (Figures 8F,H,I), consistent with previous results (Deidda et al., 2015). It is well known that GABAergic inhibition and PNN are considered plasticity brakes, and their downregulation in adulthood can effectively restore the sensitivity of the cortical network to MD (Tropea et al., 2009). However, DHF does not rescue binocular matching of orientation preference, which may be due to different threshold for ocular dominance plasticity and binocular matching. Bumetanide treatment induced a significant change in excitatory neural transmission (upregulation of GluA1, PSD-95 and spine density). DHF-induced PNN maturation could reinstate the ocular dominance plasticity but might be insufficient to completely restore the E/I balance and rescue the binocular matching.

The mechanisms underlying the heightened plasticity drives mismatching process are still unknown. One possibility is that enhancing E/I balance render the inhibitory inputs insufficient to shape the coherent neuronal output. In this scenario, the plasticity of synaptic connections and circuits during the extended critical period remains in a high level, which constantly rewire the excitatory inputs onto individual cortical neurons, so orientation preference through either eye is in an unstable state. DZ and CPP could rebalance the E/I output, thus stabilize the orientation preference through each monocular input, to rescue the binocular matching deficit. This possibility could be further investigated by *in vivo* patch-clamp to compare the EPSC and IPSC tuning through each eye, combined with chronic two-photon imaging during the extended critical period to follow the monocular orientation preference for V1 binocular neurons. In addition, a recent paper demonstrated that the binocular matching is achieved by shifting both the contralateral and ipsilateral orientation preference toward the binocular one (Chang et al., 2020), which could also apply in the binocular matching during the extended critical period.

There are several limitations in the current study. One major limitation is that we lack proper control data for the pharmacological rescue experiments. The CPP/DZ data were directly compared to the Bum data, not the Bum mice with the vehicle for CPP/DZ, which would be a more precise control because the vehicle or the injection manipulations could have an effect. However, since DZ and CPP are dissolved in saline, the chance that saline administration or cannula implantation alone would affect the normal development of visual cortex is very low, as previous literatures showed (Hensch et al., 1998; Wang et al., 2010). Another limitation is that we did not show direct evidence for the functional impairment of NKCC1 by bumetanide and the recovery by CPP/DZ. We did look at the protein expression of NKCC1, and found a decline during the development, which is independent of the bumetanide administration. This is not surprising since bumetanide is the NKCC1 antagonist, which could affect the later developmental process (Wang and Kriegstein, 2008; Liu et al., 2019), and since the expression level of NKCC1 is low around P35, the chance that CPP/DZ directly affect the NKCC1 function is low. It is interesting to ask why the P3-8 treatment of bumetanide has an effect on P35. We believe that since NKCC1 is highly expressed during this period, its blockade could have a long-term effect on the development of E/I balance, which postponed the critical period closure in the end. Homeostasis could definitely be involved, which requires further investigation.

## Conclusion

Systemic blockade of NKCC1 co-transporter with bumetanide during the period of GABA depolarization (P3-P8) may transiently prolong the critical period and postpone the binocular matching, due to abnormal heightened plasticity in the visual cortex. This deficit was accompanied by changes of synaptic properties, and can be rescued by adjusting E/I balance *in vivo*. The extended critical period alone with normal visual experience is sufficient for the completion of binocular matching in amblyopic mice. Prolonging the critical period into adulthood by knocking out Nogo-66 receptor can prevent the normal maturation of binocular matching and depth perception. This study emphasized that the proper closure of the critical period and balance between excitation and inhibition are necessary for the normal circuit formation and functional maturation during the early development.

## Data Availability Statement

The raw data supporting the conclusions of this article will be made available by the authors, without undue reservation.

## Ethics Statement

The animal study was reviewed and approved by the Animal Care and Use Committee of Fudan University.

## Author Contributions

JPC performed the experiments, ran the analysis, and wrote the first draft. XH performed the experiments and ran the analysis. QL wrote the first Python script. JHC contributed to the original idea and experimental design. YG designed the experiments and finalized the manuscript. All authors contributed to manuscript revision, read, and approved the submitted version.

## Conflict of Interest

The authors declare that the research was conducted in the absence of any commercial or financial relationships that could be construed as a potential conflict of interest.

## Publisher’s Note

All claims expressed in this article are solely those of the authors and do not necessarily represent those of their affiliated organizations, or those of the publisher, the editors and the reviewers. Any product that may be evaluated in this article, or claim that may be made by its manufacturer, is not guaranteed or endorsed by the publisher.

## Footnotes

1
https://sourceforge.net/projects/toxtrac/
